# Regulation of bone homeostasis: signaling pathways and therapeutic targets

**DOI:** 10.1002/mco2.657

**Published:** 2024-07-24

**Authors:** Zebin Wu, Wenming Li, Kunlong Jiang, Zhixiang Lin, Chen Qian, Mingzhou Wu, Yu Xia, Ning Li, Hongtao Zhang, Haixiang Xiao, Jiaxiang Bai, Dechun Geng

**Affiliations:** ^1^ Department of Orthopedics The First Affiliated Hospital of Soochow University Suzhou Jiangsu China; ^2^ Department of Orthopedics Centre for Leading Medicine and Advanced Technologies of IHM Division of Life Sciences and Medicine The First Affiliated Hospital of USTC University of Science and Technology of China Hefei China; ^3^ Department of Orthopedics Jingjiang People's Hospital Seventh Clinical Medical School of Yangzhou University Jingjiang Jiangsu Province China

**Keywords:** bone cells, bone homeostasis, signal crosstalk, signaling pathway, skeletal disease, therapeutic targets

## Abstract

As a highly dynamic tissue, bone is continuously rebuilt throughout life. Both bone formation by osteoblasts and bone resorption by osteoclasts constitute bone reconstruction homeostasis. The equilibrium of bone homeostasis is governed by many complicated signaling pathways that weave together to form an intricate network. These pathways coordinate the meticulous processes of bone formation and resorption, ensuring the structural integrity and dynamic vitality of the skeletal system. Dysregulation of the bone homeostatic regulatory signaling network contributes to the development and progression of many skeletal diseases. Significantly, imbalanced bone homeostasis further disrupts the signaling network and triggers a cascade reaction that exacerbates disease progression and engenders a deleterious cycle. Here, we summarize the influence of signaling pathways on bone homeostasis, elucidating the interplay and crosstalk among them. Additionally, we review the mechanisms underpinning bone homeostatic imbalances across diverse disease landscapes, highlighting current and prospective therapeutic targets and clinical drugs. We hope that this review will contribute to a holistic understanding of the signaling pathways and molecular mechanisms sustaining bone homeostasis, which are promising to contribute to further research on bone homeostasis and shed light on the development of targeted drugs.

## Graphical abstract

Bone homeostasis is regulated by complex signaling pathways involving osteoblasts, osteoclasts, and osteocytes, which coordinate bone formation and resorption. Disruption of this signaling network can lead to bone diseases. Additionally, the disease‐specific microenvironments in various bone conditions can further disrupt the signaling network and exacerbate disease progression. Consequently, targeting key molecules in these pathways offers promising therapeutic strategies for the treatment of skeletal diseases.

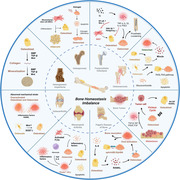

## 1 INTRODUCTION

Bone is a highly dynamic and homeostatic tissue that supports body's weight, effectively maintaining sports functions and shielding our soft internal organs, safeguarding them from external forces.^1^ To maintain skeletal health, the process of bone remodeling occurs continuously throughout an individual's lifespan. Bone remodeling is a process that involves the resorption of worn or degraded bone by osteoclasts and the replacement of new bone formed by osteoblasts.^2^ The coordinated action of bone resorption and bone formation forms the basis of bone homeostasis, which contributes to healthy bone in the adult population.

However, an imbalance between bone formation and bone resorption will disturb bone homeostasis, resulting in various diseases. For example, an overabundance of bone resorption facilitated by mature osteoclasts can result in osteoporosis, which in turn lead to fragility fractures.^3^ Collagen type I Alpha 1/Alpha 2 (COL1A1/COL1A2) mutations in osteoblasts are responsible for osteogenesis imperfecta (OI).^4^ In addition, many inflammatory conditions, such as periprosthetic osteolysis (PPO), osteoarthritis (OA),  rheumatic arthritis(RA), osteonecrosis of the femoral head(ONFH), Paget's disease of bone (PDB) and bone tumors, lead to excessive bone resorption as well as impaired bone formation.^5^ It has been approximated that more than 200 million people are impacted by osteoporosis globally,^6^ and bone loss‐related diseases have become a global burden on our aging society.^7^ Thus, maintaining homeostatic balance and ensuring the health of bone tissue are of paramount importance.

In recent decades, both fundamental and clinical studies have revealed that many signaling pathways are pivotal contributors to maintaining bone homeostasis. The maintenance of bone homeostasis is intricately governed by various signaling pathways, such as the receptor activator of nuclear factor‐kappa B (NF‐κB) ligand (RANKL)/receptor activator ofNF‐κB (RANK)/osteoprotegerin (OPG) (RANKL/RANK/OPG) signaling system, growth factor, parathyroid hormone (PTH), NF‐κB, phosphoinositide 3‐kinase AKT (PI3K/AKT), Janus kinase/signal transduction and transcription activation (JAK/STAT), mitogen‐activated protein kinase (MAPK), adenosine 5′‐monophosphate‐activated protein kinase (AMPK), Hedgehog (Hh), and Notch signaling pathways. For example, activation of the Hh signaling pathway increases the expression of RUNX Family Transcription Factor 2 (RUNX2) in osteoblastic cells.^8^ Conversely, Notch signaling serves as a suppressor of osteoblast differentiation.^9^ Fibroblast growth factor (FGF) signaling regulates preosteoblast proliferation, osteoblast fate, and the function of mature osteoblasts.^10^ Furthermore, bone morphogenetic protein (BMP) and canonical Wnt signaling induces osteoblast differentiation.^11^ Additionally, the RANKL/RANK/OPG signaling system and NF‐κB pathways, which are traditionally associated with osteoclasts, have been shown to also regulate osteoblast differentiation and function.^12^

These pathways interconnect, forming a vast and intricate network that collaborates to maintain bone homeostasis. Consequently, inhibiting only one signaling pathway might not guarantee lasting remission across all patients with bone homeostasis disorders. Several researchers are pioneering approaches for combination therapy, employing two or more agents, and are achieving promising results.^13^ Moreover, the focus on modulating intracellular pathways that target multiple shared signals has been emphasized.^14^ Therefore, it is crucial to comprehensively investigate the interrelationships between these signaling pathways and bone homeostasis. Additionally, although there are similarities in bone homeostatic imbalances in some diseases, the bone microenvironment varies in different bone disease conditions.^15^ Hence, delineating imbalances in bone homeostasis within particular microenvironmental contexts is pivotal for deciphering disease origins and advancing drug treatment. This enhanced understanding of signaling pathways across different bone homeostasis conditions has paved the way for the application of drugs from other disciplines for bone disease management, thereby broadening the spectrum of therapeutic possibilities for individuals suffering from bone loss.

This review summarizes recent advances in understanding the signaling pathways involved in bone homeostasis, including the development, differentiation, and physiological functions of osteoclasts and osteoblasts. It then concentrates on key signaling pathways that regulate bone homeostasis and highlights the pathophysiology of bone homeostasis disorders in various diseases. Based on this, we review the present therapeutic approaches in clinical use or in clinical trials that target key molecules of these signaling pathways. Finally, we discuss the remaining challenges and future research perspectives in targeting bone homeostasis. This review seeks to provide a thorough understanding of the underlying mechanisms and signaling pathways involved in bone homeostasis. This novel viewpoint on bone research has the potential to extend the current knowledge of the regulatory mechanisms of bone homeostasis and may facilitate the development of new therapeutic strategies for related disorders.

## 2 BIOLOGY OF BONE HOMEOSTASIS

The maintenance of bone homeostasis is a complex regulatory mechanism influenced by various factors and the surrounding microenvironment. Ongoing bone tissue remodeling is primarily facilitated by osteoblasts and osteoclasts,^16^ with the differentiation and function of these cell types being crucial for the upkeep of the adult skeleton through the synchronization of bone resorption and formation processes.^17^

### 2.1 Osteoblastogenesis

**FIGURE 1:**
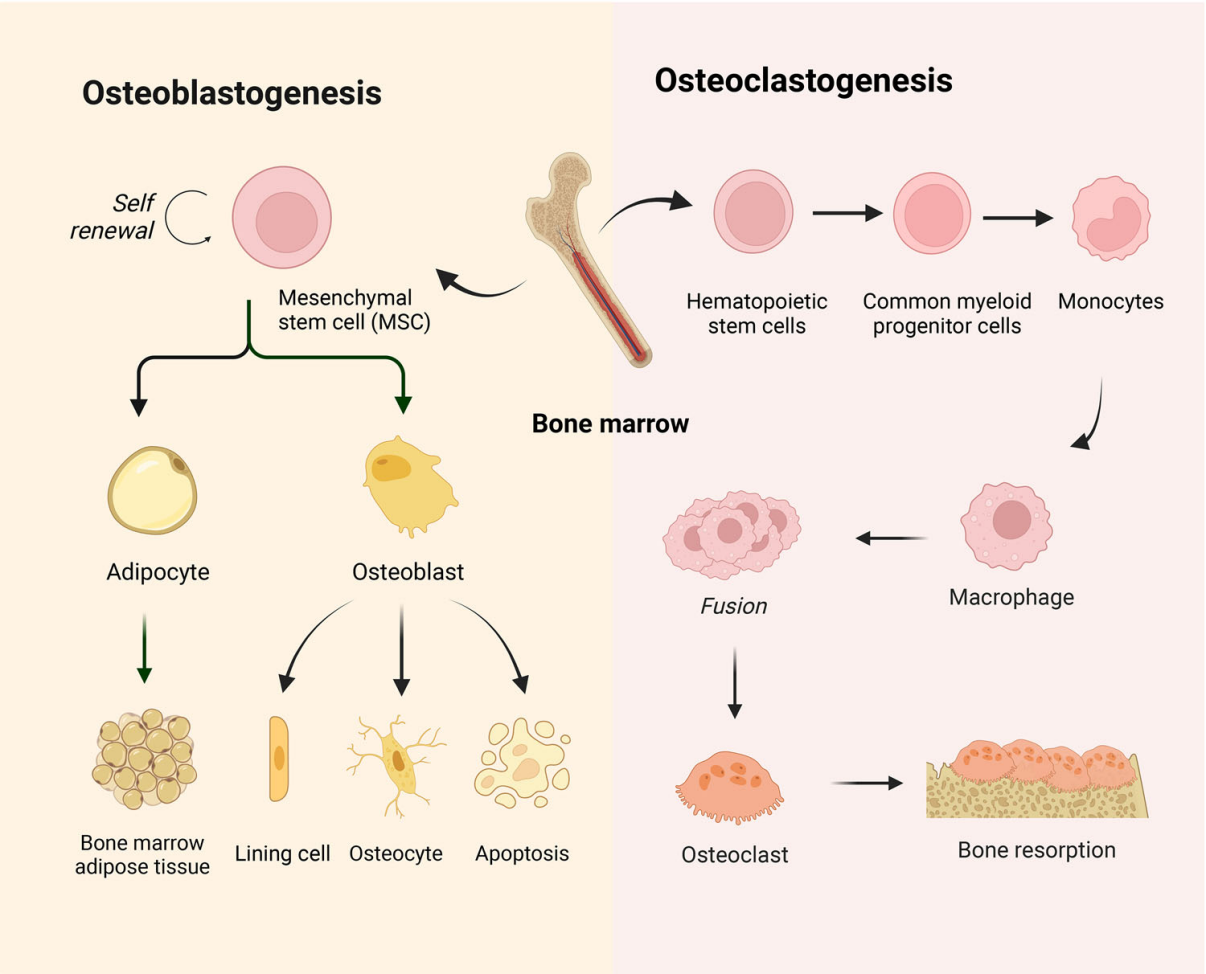
Biology of osteoblastogenesis and osteoclastogenesis. Osteoblasts originate from BMSCs, maturing through transcription factors like RUNX2 and Osterix. Osteoclasts originate from hematopoietic progenitors that give rise to monocytes and macrophages, which eventually fuse to form the multinucleated cells that resorb bone. The balanced action of these two cell types is vital for skeletal health and ensuring the continuous renewal and repair of bone tissue (created with BioRender.com).

Osteoblasts, which are derived from bone marrow mesenchymal stem cells (BMSCs), are responsible for bone formation^18^ (Figure 1). The development and differentiation of osteoblasts are regulated by a series of signaling pathways and the activation of transcription factors.^7^ Given the multilineage differentiation potential of BMSCs, their initial differentiation step involves committing to a common osteochondroprogenitor cell, triggered by the activation of key osteogenic transcription factors, including drosophila distal‐less 5 (DLX5), Osterix (OSX) and RUNX2. This commitment leads to the formation of an osteoprogenitor cell, which then progresses to a preosteoblast through the expression of initial osteogenic genes, including alkaline phosphatase (ALP) and COL1A1, and the expression of these genes is maintained throughout the lifespan of mature osteoblasts. Moreover, mature osteoblasts secrete several extracellular proteins essential for bone formation, including COL1, osteocalcin (OCN), bone sialoprotein II (BSP II), and osteopontin (OPN). The extracellular matrix (ECM), which is initially produced as a nonmineralized osteoid, eventually undergoes mineralization through the accumulation of calcium phosphate to form hydroxyapatite. After osteoblasts deposit bone where necessary, they can end up in one of three states: 60–80% of osteoblasts undergo apoptosis; transform into bone lining cells; or become osteocytes.^19^

### 2.2 Osteoclastogenesis

Osteoclasts, which originate from hematopoietic progenitors within the monocyte lineage, exhibit a distinctive capacity for bone resorption, rendering them essential for the maintenance of bone homeostasis^20^ (Figure 1). Osteoclast precursors (pOCs) are guided to the bone resorption surface from the bone marrow and circulation through sphingosine 1‐phosphate signaling. Subsequently, pOCs fuse into multinucleated osteoclasts (mOCs), which are associated with the availability of RANKL and macrophage colony‐stimulating factor (M‐CSF).^21^ Upon these stimuli, a wide array of signaling cascades, including both canonical and noncanonical NF‐κB pathways, MAPK pathways, calcium signaling pathways, and PI3K/AKT pathways, are activated.^22^ These signaling cascades mobilize a complex transcriptional network that upregulates osteoclast‐promoting factors such as NF‐κB, activator protein 1 (AP‐1), cAMP‐response element binding protein (CREB), and B‐lymphocyte‐induced maturation protein 1, and downregulating osteoclast differentiation suppressors such as interferon regulatory factor 8, MAF BZIP transcription factor B, and B‐cell lymphoma 6 (BCL‐6), which coordinately induces the expression of nuclear factors that are the “master regulators” of activated T‐cell c1 (NFATc1). As a crucial transcription factor, NFATc1 enhances osteoclast formation by increasing the transcription of genes associated with bone resorption, including matrix metalloproteinase 9 (MMP9), cathepsin K (CTSK), acid phosphatase 5, and tartrate‐resistant acid phosphatase (TRAP).^21^ In addition, mOCs are specialized cells that exhibit polarity and adhere to the bone surface, creating resorption lacuna by secreting lysosomal proteases, including MMPs, CTSK, and TRAP, in which hydrochloric acid is actively secreted, leading to the dissolution of bone mineral hydroxyapatite.^23^ In addition to RANK/RANKL and M‐CSF, other factors, such as the Wnt pathway, FGF signaling pathway, and transforming growth factor beta (TGF‐β) pathway, have been shown to participate in osteoclastogenesis.^24^

### 2.3 Osteocytes

Osteocytes, matured from OBs, are the main component of the skeleton that maintains the bone tissue environment and contributes to the osteoblasts and osteoclasts regulation during bone remodeling.^25^ Osteocytes release RANKL to induce osteoclast differentiation and OPG to inhibit osteoclast formation.^26^ In addition, osteocytes also release FGF‐23, BMP, and sclerostin (SOST) to regulate osteoblast activity. These functions largely depend on endocrine, paracrine, and autocrine mechanisms to regulate bone homeostasis.^27^ In addition, as mechanosensors of bone, osteocytes sense mechanical signals and transduce them into specific ion channel signaling.^28^ Osteocytes embed in bone tissues, forming a complex network within the bone structure and maintaining close communication with the bloodstream and other bone cells. The recently identified signaling pathway of osteocytes presents numerous possibilities for treating different bone disorders.

### 2.4 Equilibrium and communication of bone homeostasis

The equilibrium between bone anabolism and catabolism is crucial to the maintenance of bone homeostasis.^29^ Bone resorption and formation normally follow each other rather than mutually independent processes. The switch between the osteoblastic and osteoclastic phases is regulated by direct cell‐to‐cell contact.^30^ EphrinB2–EphB4 signaling involves communication between osteoblasts and osteoclasts.^31^ EphrinB2, a transmembrane protein on osteoclasts, which can bind with the receptor EphB4 on osteoblast membranes, activating signaling pathways in both osteoblasts and osteoclasts.^21^ In osteoblasts, EphB4 promotes osteogenic differentiation and suppresses osteoblast apoptosis. In osteoclasts, reversing EphrinB2 signaling can inhibit osteoclastogenesis by downregulating AP‐1 and NFATc1 expression.^32^

Furthermore, FAS (CD95) and FAS ligand (FAS–FASL) signaling has recently been shown to be involved in postmenopausal osteoporosis.^33^ FAS is a death domain‐containing member of the TNFR superfamily, and FASL mediate apoptosis upon binding to FAS. Estrogen deficiency decreases FASL expression in osteoblasts, leading to decreased osteoclast apoptosis and increased bone resorption. Estrogen deficiency decreases FASL expression in osteoblasts, resulting in reduced osteoclast apoptosis and increased bone resorption. In addition, cell vesicle communication has been shown to contribute to this transition.^2^

In addition, extracellular vesicles (EVs) are also involved in communication between osteoblasts and osteoclasts. Small osteoclast vesicles are osteoblast‐derived EVs that inhibit osteoblast differentiation by inhibiting RUNX2 and enhancing RANKL expression, which induces osteoclast differentiation.^34^ Although direct modes of conversation, such as cell‐to‐cell direct contact or cell vesicle communication between osteoblasts and osteoclasts, already exist locally to regulate bone homeostatic balance, several signaling pathways are involved in more complex bone homeostatic homeostasis, as we will discuss in the next section.

## 3 SIGNALING PATHWAYS AND MOLECULES IN BONE HOMEOSTASIS

Bone remodeling is regulated by a complex signaling pathway network. The functions of osteoblasts and osteoclasts depend on several distinct but interactive genetic programs, among which the RANKL/RANK/OPG signaling system, TGF‐β, BMP, Notch, and Wnt are associated with genetic bone diseases.^35^ Multiple signaling pathways are involved in the disease progression of bone homeostasis‐related diseases. Moreover, abnormal signals are often targets for drug discovery.

### 3.1 RANKL/RANK/OPG signaling system

The RANKL/RANK/OPG signaling system function as pivotal molecules in bone homeostasis.^21^ Produced by osteocytes, osteoblasts, and BMSCs, RANKL is a member of the tumor necrosis factor (TNF) family and is characterized as a trimeric transmembrane protein.^36^ RANK, a type I transmembrane protein, is found on osteoclast progenitors, mature osteoclasts, and some immune cells. The interaction between RANKL in osteoblasts and RANK on osteoclast progenitors triggers a series of intracellular signals via TNF receptor associated factor 6 (TRAF6), which engages pathways such as the MAPK, NF‐κB, and PI3K pathways. This cascade amplifies NFATc1 expression, fostering osteoclastogenesis and bone resorption. Interestingly, while RANK was traditionally viewed as the exclusive receptor for RANKL, research by Luo et al.^37^ revealed that leucine‐rich repeat‐containing G‐protein‐coupled receptor 4 (LGR4) is another receptor for RANKL. This receptor competes with RANK for RANKL, hindering the typical RANK signaling pathway involved in osteoclast differentiation. The interaction of RANKL with LGR4 stimulates the G protein subunit alpha Q (Gαq) and glycogen synthase kinase 3 beta (GSK‐3β) pathways, consequently diminishing NFATc1 expression and function in osteoclastogenesis.^37^

**FIGURE 2:**
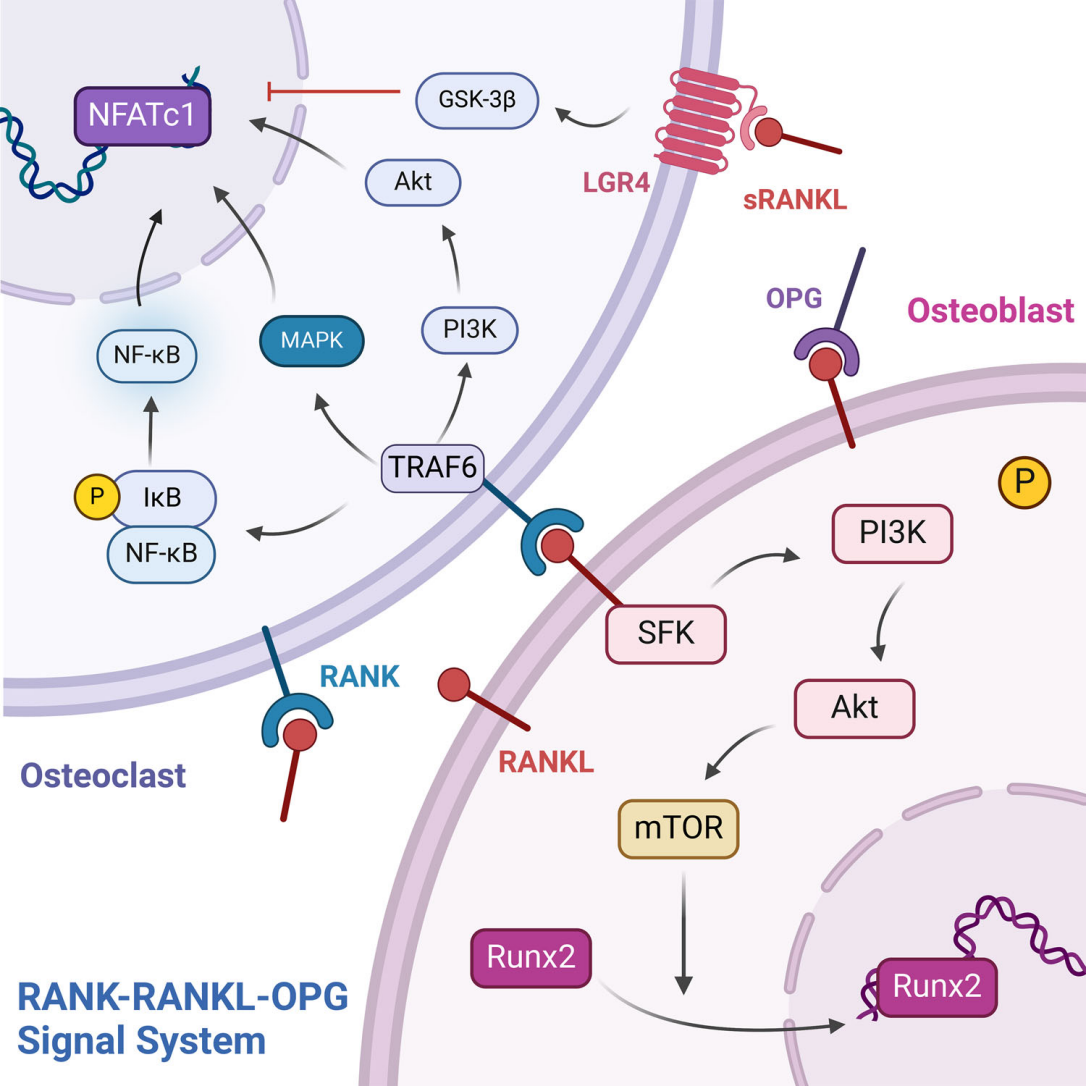
RANKL/RANK/OPG signaling system in bone homeostasis. (1) RANKL, produced by osteoblasts, binds to its receptor RANK on osteoclast progenitors, initiating intracellular signaling cascades such as MAPK, NF‐κB, and PI3K/AKT signaling pathway via TRAF6, culminating in the enhancement of NFATc1 expression. (2) RANKL binding with LGR4 suppress NFATc1 by GSK‐3β pathways. (3) Upon binding RANK, RANKL activates the PI3K/AKT/mTOR pathway and promotes nuclear translocation of RUNX2 in osteoblast. (4) OPG is a decoy receptor for RANKL and blocks RANK–RANKL binding (created with BioRender.com).

Furthermore, RANKL is also a receptor in osteoblasts and activates an intracellular “reverse” signaling pathway upon binding with RANK‐RANKL, which further promotes osteoblast differentiation and mineralization. It was suggested that the activation of the PI3K/AKT/mechanistic target of rapamycin kinase (mTOR) pathway through the interplay between RANKL and Src family kinases leads to the entry of RUNX2 into the nucleus and increases the expression of markers of early differentiation. By acting as a decoy receptor for RANKL, OPG is produced by osteoblasts and blocks the interaction between RANKL and RANK, thus preventing the formation of osteoclasts and the subsequent breakdown of bone. OPG is regulated by many factors. For example, in vitro studies have demonstrated that estrogen increases OPG production by osteoblasts,^38^ suggesting a link between estrogen withdrawal postmenopause and the development of osteoporosis due to increased OPG levels. In addition to estrogen, several signaling pathways and molecules, including cytokines such as IL‐1β, IL‐11, TNF‐α, 1α,25‐dihydroxyvitamin D3, PTH, and prostaglandin (PG), play indirect roles in bone metabolism by influencing the RANK/RANKL/OPG signaling cascade, primarily by promoting the expression of RANKL on the cell membrane, which in turn stimulates bone resorption^39^ (Figure 2).

In addition, osteocytes, the major source of RANKL, produce RANKL in a membrane‐bound and possibly soluble form and transmit RANKL signals in the bone matrix through their dendritic projections,^40^ thereby communicating with pOCs in the bone marrow and periosteal regions to further promote osteoclast differentiation and activity. Mechanical stress enhances RANKL expression in osteoblasts through the NF‐κB pathway.^41^ PTH also upregulates RANKL expression in osteoblasts. In addition, osteocytes secrete OPG, which inhibits osteoclastogenesis and bone resorption. Thus, osteocytes regulate osteoclast activity and bone resorption directly through RANKL and indirectly through OPG.^42^ In addition, osteocytes detect microdamage in bone and initiate bone remodeling through signaling. Apoptotic osteocytes in the damaged area release ATP signals that activate the release of RANKL from nearby healthy osteocytes, a process that involves the activation of pannexin‐1 (Panx1) and P2X7 receptors, which transmit signals to attract remodeling units.^43^

### 3.2 Hormone and growth factor signaling pathways

#### 3.2.1 PTH signaling pathway

PTH, the main secretory hormone, regulating calcium and phosphorus metabolism, and it is crucial for regulating bone homeostasis by influencing the activity of osteoblasts and osteoclasts.^44^ Binding of PTH to PTH‐receptor 1 (PTH1R) activates the G protein‐coupled receptor (GPCR) signaling pathway, which promotes the production of cAMP, which acts as a second messenger and activates protein kinase A (PKA) in osteoblast lineage cells. The cAMP/PKA signaling pathway increases RUNX2 and OSX activity and expression, thereby promoting the differentiation and maturation of osteoblasts. In addition, it coordinates the activity of BMP, thereby promoting bone formation.^45^ Specifically, the interaction between PTH and its receptor PTH1R triggers endocytosis of the PTH1R‐low‐density lipoprotein receptor‐related protein 6 (LRP6) complex, resulting in enhanced downstream BMP/SMAD family member 1 (SMAD1) signaling. This cascade ultimately promotes the differentiation of BMSCs into osteoblasts.^46^

PTH also indirectly activates the Wnt signaling pathway, increasing β‐catenin stability and intranuclear aggregation and promoting osteoblast differentiation. This is achieved by inhibiting the SOST, a protein that inhibits Wnt signaling, expression. Furthermore, PTH increases RANKL expression in osteoblasts and BMSCs and decreases the expression of OPG, which promotes osteoclasts survival and differentiation, enhances osteoclast activity, and indirectly results in bone resorption.^47^ Furthermore, PTH has also been shown to be associated with insulin‐like growth factor 1 (IGF‐1),^48^ which is induced by PTH in mature osteoblasts and enables PTH to induce RANKL and M‐CSF, promoting osteoclastogenesis. In addition, it has been shown that PTH exerts dual effects on bone formation and bone resorption, which depend on its mode of action.^49^ Under sustained high doses of PTH, osteoclast activity exceeds that of osteoblasts, ultimately leading to greater bone loss than bone formation.^50^

**FIGURE 3:**
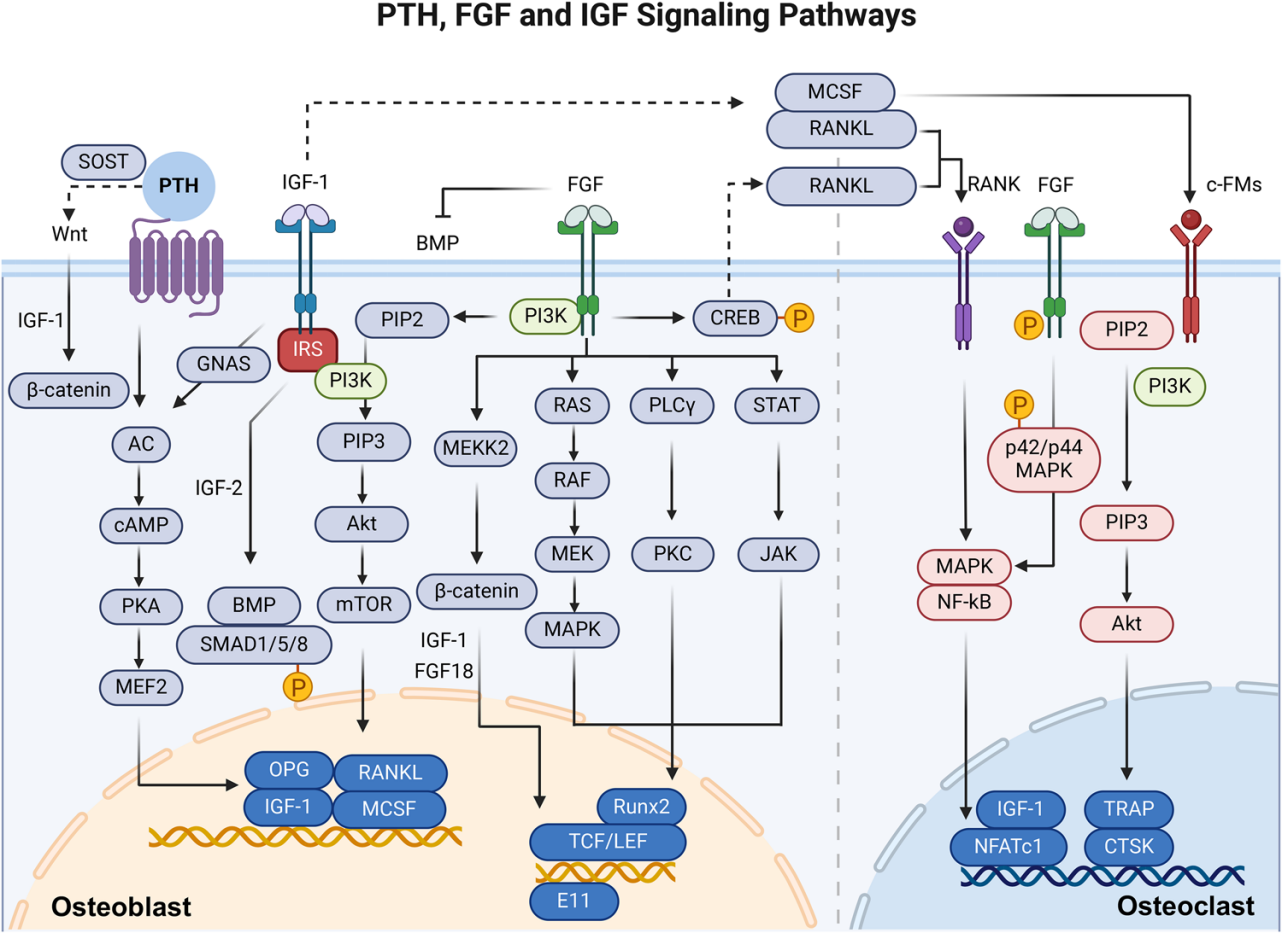
A simplified view of PTH, IGF, and FGF signaling. (1) PTH signaling: PTH activates the PTH1R receptor and then influences intracellular cAMP levels and subsequently engaging PKA and mTOR signaling, with downstream effects on osteoblast activity. (2) IGF‐1 signaling pathway: IGF‐1 signaling can promote the survival and differentiation of osteoblasts through synergistic interactions with PTH or PI3K/AKT/mTOR pathway. IGF‐2 indirectly increases the activity of BMP‐9 and promotes the nuclear translocation of SMAD1/5/8, thereby promoting the osteogenic differentiation of MSCs. (3) FGF signaling pathway: FGF can cross‐regulate the function of osteoblasts through PLCγ, JAK, PI3K, and RAS and regulate the activity of osteoclasts through MAPK pathway. (4) Indirect effect: PTH, IGF, and FGF also can indirectly regulate gene expression in osteoclast via RANK/RANKL (created with BioRender.com).

In addition to osteoblasts and osteoclasts, PTH significantly influences osteocytes. PTH targets osteocytes to regulate the expression of key genes such as SOST and RANKL. The expression of SOST, which encodes SOST, an inhibitor of bone formation, is downregulated by PTH, leading to increased bone formation. Moreover, PTH upregulates RANKL expression in osteocytes, which is essential for bone resorption by promoting osteoclast activity.^51^ Additionally, PTH affects osteocyte morphology and function, including perilacunar remodeling, which helps mobilize calcium from the bone matrix, particularly during physiological states like lactation. Osteocytes also exhibit rapid changes in response to PTH, indicating their role in immediate calcium regulation and bone remodeling dynamics^52^ (Figure 3).

#### 3.2.2 FGF signaling pathways

The FGF signaling pathway is composed of FGFs, FGF receptors (FGFRs) and downstream signaling molecules, and FGF signaling is essential for bone homeostasis by influencing the activity of osteoblasts and osteoclasts.^53^ The binding of ligands to FGFR triggers FGFR autophosphorylation, leading to the activation of docking proteins such as FGFR substrate 2 and phospholipase C‐γ (PLCγ), which in turn activate downstream pathways such as the MAPK and PI3K/AKT/mTOR pathways to regulate transcription factors.^54^

Notably, the effects of FGF/FGFR signaling on osteoblastogenesis are complex because they are dependent on the type of FGF and the FGFR expressed and the stage of cell differentiation.^55^ In mesenchymal progenitor cells, FGFR1/2 are important for maintaining BMSCs by inhibiting cellular senescence.^56^ During osteogenic differentiation, FGF‐2 predominantly stimulated the ERK signaling pathway in cells resembling osteoblasts. In more mature osteoblasts, increased FGFR2, FGFR3, and FGFR4 expression and decreased FGFR1 expression correlate with an inability to maintain FGF2 responses.^57^ FGF‐2 signaling engages the inositol polyphosphate cascade, which involves inositol hexakisphosphate kinase, a regulator of RUNX2 and osteoblast gene expression.^58^ Moreover, E11/podoplanin is important in the early stages of osteoblast‐to‐osteocyte transformation, and FGF‐2 enhances the expression of E11 or promotes the acquisition of an osteocyte phenotype.^59^ Furthermore, other FGFs, including 8, 9, 18, and 23, are mainly associated with osteoblast differentiation and bone regeneration.^60^ FGF‐8 stimulates osteoblast proliferation via a MAPK‐independent pathway.^61^ FGF‐9 expressed in the periosteum and surrounding tissues also interacts with FGFR1 and FGFR2. FGF‐18 stimulates the PI3K/ERK pathway and BMP‐2/SMAD pathway by blocking noggin, a BMP‐2 antagonist that is essential for bone formation.^62^, ^63^ FGF‐23 indirectly regulates OPN secretion by reducing ALP transcription and phosphate generation in osteoblasts, functioning through FGFR3 without Klotho.^64^ Similarly, FGFR1 signaling is required for osteocyte survival. Mice lacking FGFR1 in osteoblasts exhibit heightened β‐cyclin signaling and elevated trabecular bone density, resulting from enhanced bone formation and reduced bone resorption. FGFR1 suppresses β‐catenin expression and diminishes the activity of Wnt/β‐catenin.^65^, ^66^ In summary, these pathways activated by the FGF/FGFR signaling pathway control osteoblast formation and osteocyte survival.

Different FGFs/FGFRs also have different effects on osteoclasts. FGF‐2 directly targets osteoclasts through FGFRs such as FGFR1 and FGFR3 to promote bone resorption.^67^ FGF‐2 can activate autophosphorylation of FGFR1 and subsequent phosphorylation of the p42/p44 to activate the MAPK pathway.^68^ Additionally, CREB phosphorylation is activated by FGF‐2, enhancing the association of p‐CREB with the promoter region of the RANKL gene.^69^ In addition, FGFR3 also participates in the regulatory role of FGF‐2 in bone resorption, boosting osteoclast adherence and elevating osteoclastic enzyme levels related to resorption.^70^ FGF‐6 prompts pOC fusion into larger cells by activating MAPK signaling pathways induced by RANKL.^71^ FGF‐18 can also indirectly induce osteoclast formation by stimulating the expression of RANKL and osteoblast cyclooxygenase‐2.^72^ However, FGF‐8a inhibits osteoclastogenesis via a RANKL/OPG‐independent mechanism.^61^

Similarly, FGFs play a significant role in osteocyte function and morphology.^73^ FGF‐7 enhances the expression of connexin 43 (Cx43) in osteocytes, which is crucial for gap junction formation, thus facilitating cell‐to‐cell communication. This regulation is mediated through the β‐catenin signaling pathway, where FGF‐7 induces the accumulation and nuclear translocation of beta‐catenin, promoting the formation and elongation of osteocyte cell processes.^74^ These changes help osteocytes maintain their interconnected network, which is crucial for sensing mechanical and hormonal signals and regulating bone remodeling​. (Figure 3)

#### 3.2.3 IGF signaling pathways

IGF, which is genetically structured with high homology to insulinogen, regulates a variety of biological activities.^75^ The IGF signaling pathway is composed of two ligands (IGF‐1 and IGF‐2), two corresponding receptors (IGFR1 and IGFR2), and six IGF‐binding proteins (IGFBP‐1 to 6) with high affinity for IGF.^76^ Among them, IGF‐1 is the primary ligand and binds to IGFR1 with 20‐fold greater affinity than does IGF‐2, which hardly binds to IGFR2.^77^ IGFR1, a receptor possessing tyrosine kinase activity, exhibits a significant degree of similarity to the insulin receptor,^78^ whereas IGFR2 is a single‐channel transmembrane receptor without kinase activity.^79^ In addition, IGFBP is an endocrine regulator of IGF activity.^80^ The IGF system is present in many human tissues, and it promotes cell proliferation and differentiation through paracrine, endocrine, and autocrine mechanisms.^81^ When IGF‐1 binds to IGFR1, it initiates downstream signal transduction pathways, including the RAS/RAF/MEK/ERK and PI3K/AKT/mTOR signaling pathways, to induce IGF‐1‐mediated functions.^77^

IGF‐1 signaling plays a crucial role in the proper development and functioning of osteoblasts by promoting the viability, growth, specialization, and ECM synthesis of cultured osteoblast cells.^82^ IGF‐1 can stimulate the osteogenic differentiation of osteoblast precursors through the PI3K/AKT/mTOR pathway.^83^ IGF‐1 can induces osteogenic differentiation of BMSCs and maintains bone microarchitecture and bone quality by activates mTOR.^84^ In addition, IGF‐1 signaling directly enhances osteoblast differentiation and indirectly enhances osteoblast differentiation through the stabilizing effect of IGF‐1 on β‐catenin.^85^ Furthermore, IGF‐1 interacts with PTH, inducing the activation of downstream signaling molecules, thereby promoting osteoblast differentiation and maturation.^46^ PTH stimulates osteoblast formation by activating the adenylyl cyclase/cAMP/PKA signaling pathway.^86^ IGF‐1 promotes osteoclast differentiation and maturation mainly by stimulating the expression of RANKL and M‐CSF in osteoblasts.^87^, ^88^, ^89^ IGF‐2 increases the activity of BMP‐9 and facilitates the nuclear translocation of SMAD1/5/8 via the PI3K/AKT signaling pathway, which enhances the osteogenic differentiation of BMSCs.^90^ Moreover, reprogramming from glycolysis to oxidative phosphorylation (OxPhos) in chondrocytes is critical for long bone growth. IGF‐2 maintains the balance between glycolysis and OxPhos during chondrocyte maturation. This careful regulation precisely controls maturation from proliferation to hypertrophy during endochondral ossification.^7^

In conclusion, IGF‐1 is mostly derived from osteocytes and acts as an important mediator of bone mechanosensitivity, influencing the osteogenic response to mechanical loading. IGF‐1 expression in osteocytes is upregulated in response to mechanical strain, facilitates communication with other osteocytes, promotes bone formation and inhibits bone resorption.^91^ In addition, osteocyte‐derived IGF‐1 is essential for developmental bone growth and contributes to the regulation of bone turnover in response to mechanical stimuli and calcium stress.^92^ (Figure 3)

#### 3.2.4 BMP signaling pathways

BMP, a member of the TGF‐β superfamily, plays a crucial role in the control of bone formation, embryonic development and various other physiological processes.^93^ It has been identified that over 20 BMP family members exist in humans, and they have diverse functions in regulating cellular processes. BMP signaling can be categorized simply into SMAD‐dependent and SMAD‐independent pathways.^94^ BMPs interact with homomeric type II receptors, which leads to the transphosphorylation of homomeric type I receptors and triggers both SMAD‐dependent and non‐SMAD‐dependent signaling pathways.^95^

In SMAD‐dependent signaling, phosphorylated SMAD (SMAD1, 5, or 8) is complexed with SMAD4 and cotransported into the nucleus to regulate the expression of osteogenic genes such as RUNX2, DLX5, myocyte enhancer factor 2c, old astrocyte specifically induced substance, KLF transcription factor 4, osteomodulin (OMD), and OSX in osteoblasts.^96^ In addition, studies have demonstrated that RUNX2 and OMD are influenced not only by the BMP signaling pathway, which is a downstream target of BMP but also regulate the expression of BMPs reversely. RUNX2 regulates the BMP4 pathway by inhibiting the transcription of chordin like 1, an antagonist of BMPs.^97^ OMD can promote BMP/SMAD signal transduction through binding to BMP‐2 and its membrane receptors. Thus, RUNX2 and OMD create a positive feedback loop in the BMP/SMAD signaling pathway.^98^ In the non‐SMAD‐dependent pathway, activated TGFβ‐activated kinase 1 (TAK1) recruits TAK1‐binding protein 1 to trigger the MKK–ERK1/2 or MKK–p38 MAPK signaling cascade. This cascade results in the phosphorylation of the transcription factors RUNX2, DLX5, and OSX, enhancing their transcriptional activity.^99^

**FIGURE 4:**
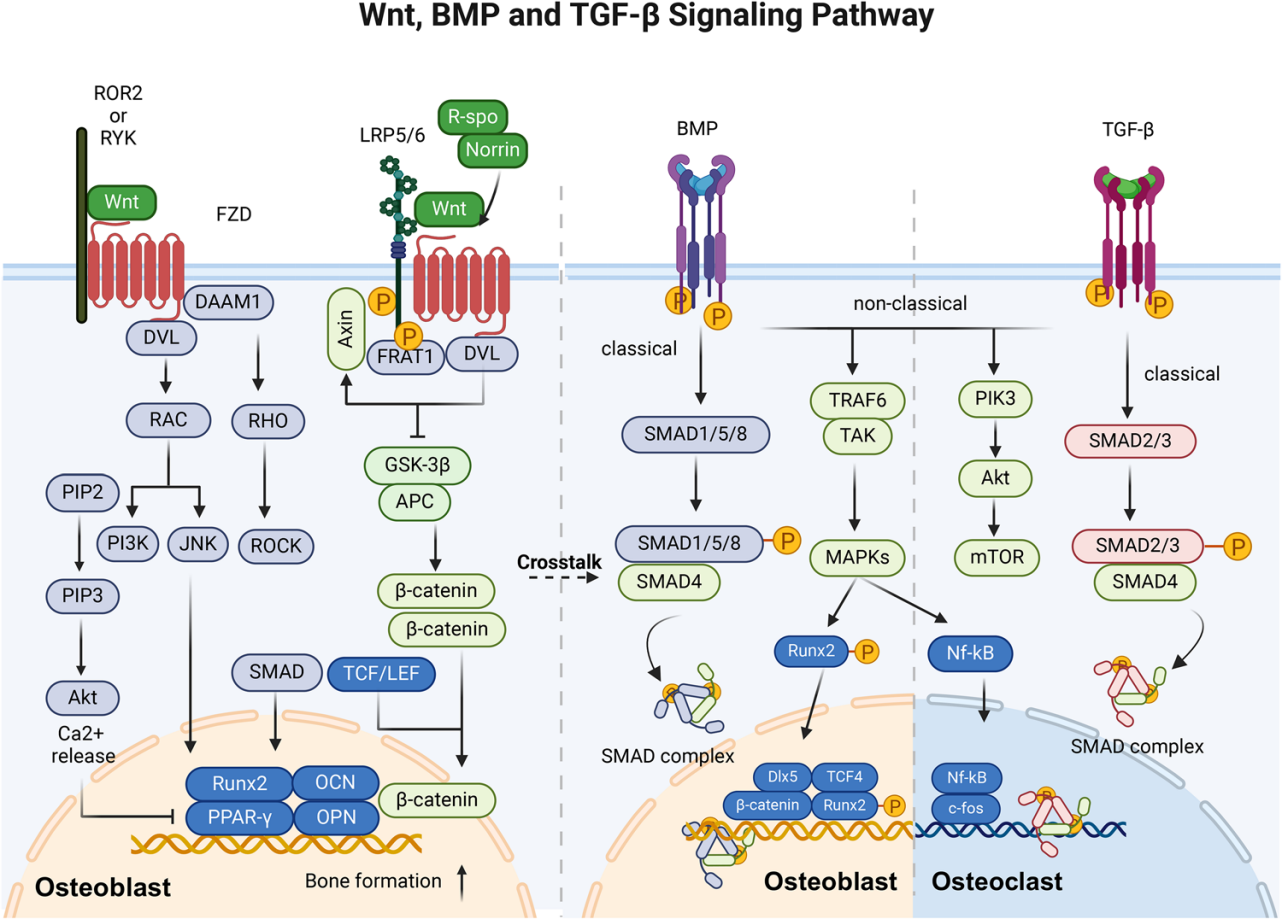
Overview of Wnt, BMP, and TGF‐β signaling pathways. (1) In the active Wnt signaling pathway, Wnt ligands bind to FZD and LRP5/6 receptors, leading to GSK‐3β degradation. β‐catenin enters the nucleus to drive gene transcription and can indirectly regulate the RANKL/RANK and BMP signaling pathways. In the inhibited Wnt signaling pathway, β‐catenin is degraded by protein complexes, resulting in ubiquitin‐mediated proteolysis and no gene transcription. (2) BMP interacts with type II receptors, leading to phosphorylation and activation of R‐SMADs (SMAD1, 5, 8), which then complex with SMAD4 and are cotransported into the nucleus to regulate the expression of osteogenic genes such as RUNX2 and DLX5 in osteoblasts. (3) TGF‐β binds to its receptors, leading to the activation of SMAD2/3, which then associates with Smad4. This complex enters the nucleus and affects the expression of genes that regulate osteoclasts and osteoblasts. (4) In the nonclassical pathway, BMP activates TRAF6/TAK1, triggering MAPK pathways and leading to the phosphorylation of transcription factors like RUNX2, promoting bone formation. TGF‐β can also activate downstream factors like MAPK and PI3K, which regulate transcription factors for bone formation and resorption (created with BioRender.com).

Among the 14 types of BMPs, BMP‐2, BMP‐4, BMP‐5, BMP‐6, and BMP‐7 demonstrate significant osteogenic properties. Conversely, BMP‐3, which is primarily produced by osteoblasts and osteocytes, is a “noncanonical” BMP variant that triggers SMAD2/3 activation to counteract the osteogenic effects associated with other BMPs.^100^ BMP also promotes the osteogenic program by affecting the Wnt signaling pathway. BMP‐2 induces an increase in the LDL receptor‐related protein 5 (LRP5) level and suppresses the expression of beta‐transducin repeat containing e3 ubiquitin protein ligase that facilitates the degradation of β‐catenin. This process leads to the stabilization of β‐catenin and activation of the canonical Wnt pathway, ultimately facilitating osteogenic differentiation.^101^ Moreover, the SMAD complex can physically interact with the transcription factor 4 (TCF4)/β‐catenin complex at specific DNA‐binding sites.^102^ Deletion of Smad4 leads to the cleavage of β‐catenin and a reduction in the expression of the LRP5.^103^ LGR4 is stimulated by BMP2, which is an orphan receptor and regulator of the Wnt pathway.^104^ In addition, the BMP inhibitor dorsomorphin hinders osteoclast formation and bone resorption.^105^ At the molecular level, BMP signaling facilitates the upregulation or enhancement of osteoblast associate transcription factors. BMP receptor type II (BMPRII) interacts with RANK and activates both p‐SMAD1/5/8 and NF‐κB signaling.^106^ In addition, BMP may be essential for promoting osteoblast‐osteoclast coupling in bone, and BMP can promote osteoclast formation by increasing the RANKL/OPG ratio.^107^ In conclusion, BMP signaling can promote osteoclast differentiation through direct and indirect pathways. (Figure 4)

#### 3.2.5 TGF‐β signaling pathway

TGF‐β is crucial for bone metabolism by intricately influencing the functions of osteoclasts and osteoblasts, primarily through the activation of SMAD signaling pathways, which in turn regulate critical transcription factors.^108^ The TGF‐β superfamily consists of three subfamilies: activin, TGF‐β and BMP.^109^ In the classical signaling pathway, TGF‐β‐specific receptor‐regulated SMAD (SMAD2/3) is activated through phosphorylation at the Ser–Ser–X–Ser structural domain and forms a complex with SMAD4. Subsequently, the activated SMAD complex is regulated by the LEM domain nuclear envelope protein and translocates to the nucleus, where it modulates the transcription of specific target genes.^110^, ^111^

According to previous studies, the TGF‐β signaling pathway is important in bone remodeling in a stage‐dependent manner and through interactions with other pathways.^112^ In the early stage of osteoblast proliferation and differentiation, TGF‐β initiates the osteogenic process by promoting the proliferation of osteoprogenitor cells.^113^ It does this by activating the SMAD2/3 signaling pathway. Once activated, SMAD2 and SMAD3 translocate to the nucleus, where they influence the transcription of key osteogenic genes. For instance, through SMAD activation, TGF‐β upregulates the RUNX2 expression, which is a master transcription factor critical for osteoblast differentiation. The activated SMAD proteins directly interact with RUNX2, enhancing its transcriptional activity and promoting the expression of genes essential for osteoblast maturation and function.^114^ However, at further stages of maturation, it inhibits osteoblast maturation, mineralization, and conversion to osteocytes.^115^ In addition, TGF‐β also exerts regulatory effects on osteoblasts through its interaction with other pathways, such as the Hh and Wnt pathways, providing a complex regulatory network for bone formation. For example, the TGF‐β‐induced activation of SMADs can intersect with Wnt signaling, further modulating Runx2 activity and osteoblast differentiation.^116^

While TGF‐β directly promotes osteoblast function, it indirectly affects osteoclasts by modulating the expression of RANKL and OPG, key regulators of osteoclast differentiation and activity. This modulation is believed to occur through TGF‐β‐induced changes in osteoblasts and stromal cells that, in turn, alter their expression of RANKL and OPG, thus impacting osteoclasts.^117^ However, a continuous increase in TGF‐β downregulated the expression of RANK and weakened the RANK‐RANKL signaling pathway, thereby inhibiting the generation of osteoclasts.^118^

In conclusion, TGF‐β orchestrates a sophisticated regulatory network that balances bone formation and resorption, acting through the SMAD signaling pathway to control the activity of key transcription factors such as RUNX2. Ultimately, TGF‐β achieves stage‐specific regulation that promotes the early differentiation of osteoblasts and osteoclasts and limits their late maturation.^119^ (Figure 4)

### 3.3 Wnt signaling pathway

The Wnt signaling pathway is evolutionarily preserved and controls the maintenance of tissue homeostasis. The Wnt signaling pathway can be categorized into two main pathways: the canonical pathway (also termed the Wnt/β‐catenin pathway), which relies on the activity of β‐catenin, and the noncanonical pathway (consisting of the Wnt/PCP pathway and Wnt/Ca^2+^ pathway), which operates independently of the activity of β‐catenin.^120^ In the Wnt/β‐catenin signaling pathway, upon binding of Wnt ligands to frizzled class receptor (FZD) and LRP5/6, the multiprotein complex known as the β‐catenin “destruction complex” is mobilized to the cellular membrane through its interaction with FZD. Consequently, the complex undergoes a loss of function in degrading β‐catenin. Subsequently, β‐catenin is translocated to the nucleus where it facilitates the activation of target genes by engaging with T‐cell factor/lymphoid enhancer‐binding factor (TCF/LEF).^121^ The canonical Wnt signaling pathway is crucial for osteoblast differentiation, bone development, bone homeostasis, and bone remodeling in living organisms.^122^ When different types of Wnt ligands (such as Wnt‐1, Wnt‐3a, Wnt‐10a, and Wnt‐10b^123^) interact with receptors on the cell membrane, they initiate downstream gene expression and determine the specificity of the Wnt pathway.^116^ Traditionally, Wnt‐10a, Wnt‐10b, Wnt‐1, and Wnt‐6 suppress the differentiation of BMSCs to adipocytes and facilitate the differentiation of BMSCs to osteoblasts through the canonical Wnt pathway.^124^ Wnt‐3a, Wnt‐4, and Wnt‐7b activate osteoblast differentiation and mineralization through the Gαq/phospholipase C‐β/protein kinase c delta (Gαq/11/PLCβ/PKCδ) pathway.^7^ Wnt‐7a modulates osteoblast proliferation by influencing RUNX2 expression.^125^ These Wnt ligands collectively establish a regulatory framework indispensable for the proper formation and functionality of osteoblasts.

According to traditional views, the Wnt signaling pathway also exerts a significant influence on energy metabolism within osteoblasts. Wnt/β‐catenin regulates fatty acid β‐oxidation through the activation of β‐catenin, which is dependent on the activation of mTORC2 and mTORC1. These factors in turn regulate the use of glucose and glutamine, respectively.^126^

In addition, extensive evidence supports the interplay between the Wnt signaling pathway and other signaling pathways, such as the BMP, TGF‐β, FGF, Hh, Notch, and PDGF pathways, in regulating the gene network responsible for controlling osteoblast differentiation and bone formation.^116^ Activation of the canonical pathway leads to the transcription of BMP‐2.^127^ BMP‐2 serves as a significant autocrine and paracrine growth factor, and its TCF/LEF‐responsive component governs signals that initiate specific transcriptional programs necessary for bone formation. This mechanism facilitates the differentiation of mesenchymal precursor cells into mature osteoblasts.^128^ Furthermore, stabilized β‐catenin can enhance the activation of BMP‐9‐induced ALP and the expression of OCN and OPN in osteoblasts.^129^ Similarly, the noncanonical pathways also play crucial roles in osteocyte migration and osteoblast differentiation.^7^ Wnt‐1 accelerates bone fracture healing and enhances bone formation by activating the yes‐associated protein (YAP)/BMP signaling pathway.^130^ Wnt5a was shown to promote BMP‐2‐mediated osteoblast differentiation via a SMAD‐independent pathway by activating nonclassical Wnt signaling through ROR2.^131^ Furthermore, the Wnt/β‐catenin signaling pathway in osteoblasts and osteocytes indirectly inhibits the differentiation of osteoclasts and the resorption of bone by promoting the secretion of OPG.^132^

In osteoclasts, the Wnt pathway has been shown to indirectly influence osteoclast formation by controlling the production of RANKL and OPG under normal physiological conditions.^133^ Primarily, the classical pathway plays a pivotal role in osteoclastogenesis via β‐catenin, which has varying impacts at different developmental stages. The activation of β‐catenin in early precursors promotes their proliferation, yet at advanced stages, it impedes osteoclast formation.^134^ In contrast to the spatial and temporal regulation of β‐catenin by the nonclassical pathway, the nonclassical pathway has been shown to promote the differentiation of pOCs.^135^ It also plays a crucial role in blocking NFATc1 transcription factor activation by RANKL. The administration of Wnt‐3a triggers a rapid increase in cAMP levels, which leads to the phosphorylation and subsequent activation of PKA, culminating in the increased phosphorylation (and thus inactivation) of NFATc1^136^ (Figure 4).

### 3.4 NF‐κB signaling pathway

The NF‐κB family, encompassing transcription factors crucial for skeletal cell differentiation, is pivotal for skeletal health.^137^, ^138^ The NF‐κB signaling pathway can be categorized as the canonical and noncanonical pathways. In the canonical pathway, upon exposure to RANKL, TNF‐α, IGF signaling, IκB kinase β (IKKβ) and IκB kinase γ (IKKγ, also known as NEMO) phosphorylate and degrade IκB kinase α (IKKα), resulting in the nuclear translocation of the NF‐κB1 (p50) and RelA (p65) complex.^139^ Conversely, in the noncanonical route, cytokine stimulation leads to TNF receptor associated factor 3 (TRAF3) degradation after binding to the cytokine receptor, stabilizing NF‐κB‐inducing kinase (NIK), continuously degraded through the TRAF3 interaction. This stabilization activates IKKα, prompting the conversion of NF‐κB2 (p100) into its active RelB (p52) form and enabling RelB/p52 complexes to enter the nucleus.^140^ However, not all TNF family cytokines can initiate this pathway; RANKL does, but TNF‐α does not.^141^

In osteoclasts, activation of both pathways is instrumental for differentiation, longevity, and functionality.^142^ Mice lacking NF‐κB p50 and p52 exhibit notable dwarfism, thickened hypertrophic chondrocyte layers, severe osteopetrosis, and a lack of osteoclasts.^143^ Whereas NF‐κB activation in osteoblasts can either stimulate or inhibit osteogenesis. For example, TNF‐induced NF‐κB activation suppresses bone formation by obstructing RUNX2, thus interfering with BMP signaling.^144^ The activity of the IKK complex also hampers osteoblast functionality. A study involving IKKalpha knockdown in OSX^+^ cells reported no impact on osteolineage cells.^145^ Conversely, inhibiting NIK increases p100 levels, enhancing bone creation and osteoblast count, while disrupting RelB bolsters bone formation.^146^ Early osteogenesis is promoted by NF‐κB activation, and p65/RelA is crucial for osteoblast functionality and survival. At low doses, TNF‐α can promote osteogenesis through NF‐κB activation.^147^ These findings highlight that the role of NF‐κB in osteoblast differentiation is complex and dependent on context.

Crosstalk between the noncanonical NF‐κB pathway and other signaling pathways also plays an important regulatory role in osteoblasts and osteoclasts. TRAF3 serves to restrict the activation of GSK‐3β induced by TGF‐β1 (via phosphorylation at Tyr216) and the degradation of β‐catenin in mesenchymal progenitor cells. This mechanism enables β‐catenin to sustain osteoblast differentiation and promote the expression of OPG, thereby mitigating bone degradation. RelA and RelB enhance osteoblastic RANKL expression, promoting osteoclastogenesis and a self‐sustaining cycle of bone destruction, TGF‐β release, TRAF3 degradation, and NF‐κB activation.^148^

**FIGURE 5:**
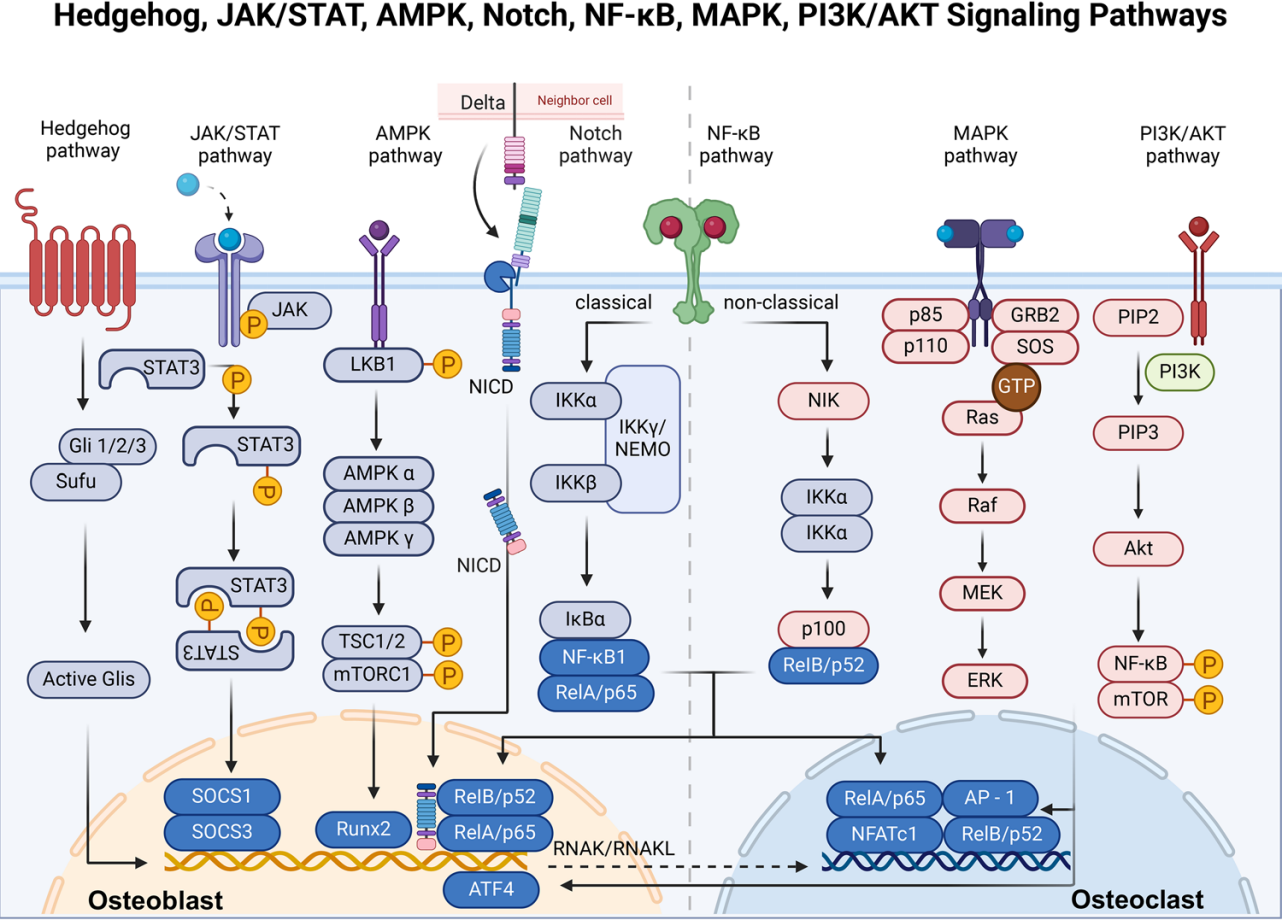
A simplified view of Hedgehog, JAK/STAT, AMPK, Notch, NF‐κB, MAPK, and PI3K/AKT pathways. (1) Hedgehog pathway: Hh proteins relieve the inhibitory effect of Ptch on Smo by binding to Ptch on target cells, leading to Smo activation, followed by further activation of Glis and regulation of downstream target genes. (2) JAK/STAT pathway: JAK/STAT pathway is activated by IL‐6, leading to the phosphorylation of STAT3, which then impacts gene expression related to osteoblast activity. (3) AMPK pathway activation through LKB1 phosphorylation inhibits mTORC1 via TSC1/2, affecting osteoblast function. (4) Notch pathway: binding of ligands expressed by neighboring cells to Notch receptors results in the release of NICD from the membrane and translocation to the nucleus, thereby activating transcription of specific genes. (5) NF‐κB pathway: divided into classical and nonclassical branches, shows how different stimuli lead to the activation of distinct NF‐κB subunits, which then translocate to the nucleus influencing both osteoclast and osteoblast gene expression. (6) MAPK pathway: MAPK is activated by various extracellular signals leading to the sequential activation of MAPKKK and MAPKK, further activating downstream molecules such as ERK, JNK, and p38 MAPK. (7) PI3K/AKT pathway: activation of PIP 3 by binding of activated PI3K to PIP2 leads to Akt phosphorylation, linking to NF‐κB and mTOR signaling, pivotal for osteoclast survival and function (created with BioRender.com).

In osteocytes, NF‐κB responds to mechanical stress by mediating the cellular response to bone loading and unloading.^149^ Mechanical stress induces the activation of NF‐κB in osteocytes, leading to the expression of genes such as RANKL, which is crucial for osteoclastogenesis. This process facilitates communication between osteocytes and other bone cells, coordinating bone remodeling. NF‐κB activation in osteocytes also plays a role in regulating bone resorption and formation and maintaining bone homeostasis.^41^ Additionally, the interaction of NF‐κB with other signaling molecules in osteocytes is critical for adapting bone structure to mechanical demands^150^ (Figure 5).

### 3.5 PI3K/AKT signaling pathway

The PI3K/AKT signaling pathway regulates cellular biological processes, such as cell proliferation and metabolism, by activating PI3K and causing AKT phosphorylation.^151^ The major components of the PI3K/AKT signaling pathway include PI3K family and the AKT family (also known as protein kinase B family; PKB).^152^ PI3Ks are an important class of kinases that include three classes of molecules (I, II, III).^153^ PI3K Class I includes two isoforms (IA and IB), which use phosphatidylinositol (PI), phosphatidylinositol‐4‐phosphate (PIP), and phosphatidylinositol‐4,5‐bisphosphate (PIP2) as substrates, phosphorylating inositol ring position 3 of the substrate. PI3K Class I can be activated by both receptor tyrosine kinases (RTKs) and non‐RTKs on the cell surface.^154^ PI3K Class II has a C2 structural domain and mainly phosphorylates PI and PIP,^155^ while Class III involves vacuolar protein sorting 34 homolog (Vps34) and p150, targeting PI and primarily concerned with cell growth and survival regulation.^156^ AKT, a serine/threonine kinase, consists of approximately 480 amino acid residues, which is one of the major downstream effector molecules of PI3K and can be involved in the regulation of a variety of life activity processes by directly phosphorylating various transcription factors, such as NF‐κB and mTOR.^157^ The AKT molecule consists of a short carboxy‐terminal regulatory domain, a PH domain and a central catalytic domain.^158^ Among them, the PH structural domain mediates the process of membrane translocation after AKT activation^159^; the catalytic structural domain contains an ATP‐binding site^160^; and the C‐terminal regulatory domain is proline‐rich and contains another phosphorylation site, Ser473.^161^ AKT family members include AKT1/PKBα, AKT2/PKBβ, and AKT3/PKBγ.^162^

The PI3K/AKT signaling pathway is regulated by various factors, including RTK and PI3K for upstream regulation^163^; phosphatase and tensin homolog and PKB for internal regulation^164^; and mTORC1, GSK3, and BCL‐2 for downstream regulation.^165^ In recent years, studies have supported the involvement of the PI3K/AKT pathway in regulating bone homeostasis through various cells.^166^ In osteoblasts, PI3K inhibits osteoblast apoptosis through the activation of AKT and further activates PI3K/AKT through Wnt‐3a.^167^ The PI3K/AKT pathway also plays an important role in osteoclast formation. Recently, several researchers have reported that Akt activation can limit osteoclast differentiation by activating the GSK‐3β/NFATc1 signaling cascade.^168^ Moreover, Karkache et al.^169^ reported that AKT, a downstream target of PI3K, can mediate the proliferation and survival of RANKL‐ and/or M‐CSF‐stimulated osteoclast lineage cells. Similarly, Wang et al.^170^ demonstrated that the PI3K/AKT signaling pathway is closely related to bone homeostasis through a network pharmacological study of the antiosteoporotic effect of dulcolax cyclen ether terpene glycosides, which is also in line with previously reported results. In conclusion, the PI3K/AKT signaling pathway can promote osteoclast differentiation and facilitate osteoblast apoptosis.

In addition, the PI3K signaling pathway in osteocytes is essential for their survival and function, particularly in response to mechanical loading. Mechanical stress activates the PI3K/Akt pathway in osteocytes, promoting their survival by inhibiting apoptosis.^171^ This activation also enhances the release of signaling molecules such as BMP‐7, which protects osteocytes from glucocorticoid‐induced apoptosis^172^ (Figure 5).

### 3.6 JAK/STAT signaling pathway

The JAK/STAT signaling pathway is involved in cell proliferation, differentiation, survival, and apoptosis and mediates immune dysregulation and tumorigenesis in organisms.^173^ It consists of ligand‒receptor complexes, the JAK family and the STAT family.^174^ Canonical JAK/STAT signaling is activated when cytokines (e.g., interferon, IL, etc.) bind to the plasma membrane receptor. It triggers a conformational change in the receptor and leads to dimerization, which further activates the activity of JAK.^175^ Activated JAK further phosphorylates the receptor to create a docking site for STAT. JAK‐phosphorylated STAT detaches, forming homodimers or heterodimers through SH2 domain‐phosphotyrosine interactions, which then regulate target gene transcription.^176^ In addition, previous studies have demonstrated that the JAK/STAT signaling pathway is also involved in complex nonclassical signaling pathways, interconjugating with other signaling pathways, such as the MAPK/ERK and PI3K/AKT/mTOR pathways, to mediate a series of biological effects.^177^ Increasing evidence indicates that JAKs and STATs are responsible for signaling via multiple hormones, growth factors, and cytokines.^178^ Therefore, they are associated with the proliferation and differentiation of osteoblasts and osteoclasts, which is crucial for skeletal development and bone homeostasis.^179^

First, osteoclast formation is usually stimulated indirectly through multiple pathways via the actions of JAK1 and STAT3 in inflammatory and subsidiary cells.^180^ Additionally, osteoblast differentiation is secondary to stimulated osteoclast formation through IL‐6 family cytokines.^181^ IL‐6 can activate STAT3 signaling within osteoblasts, leading to the upregulation of various factors, including RANKL and C‐X‐C motif chemokine ligand 1. These factors play a crucial role in initiating pOC cells to differentiate into osteoclasts and initiate the process of bone resorption.^182^ In addition, STAT3 signaling also promotes bone formation through direct signaling in osteocytes, mainly by stimulating the expression of transcription factors (e.g., C/EBPδ and C/EBPβ) to induce osteoblast differentiation.^183^ In conclusion, normal bone remodeling and bone homeostasis depend on the JAK1/STAT3/suppressor of cytokine signaling 3 (SOCS3) signaling pathway.^180^ Moreover, the JAK2/STAT5B pathway is also fundamental for osteoblast formation,^184^ as it acts on important transcription factors, such as T box transcription factor 3, RUNX2, and BMP‐7,^185^ which further illustrates the role of JAKs and STATs in bone conversion under inflammatory conditions. JAK inhibition increases the expression of OCN and the Wnt signaling pathway (by stabilizing β‐catenin).^186^

Furthermore, osteocytes are regulated by factors such as LIF and subsequent activation of LIFR, which are modulated by the JAK/STAT pathway. This pathway supports the expression of SOST and other osteocyte‐specific genes that control bone mineral density and structural integrity. Through miR‐30 and its effect on RUNX2, the pathway indirectly influences osteocyte maturation and function, highlighting its critical role in skeletal biology and disease processes such as osteoporosis^187^, ^188^ (Figure 5).

### 3.7 MAPK signaling pathway

MAPK, a member of the Ser/Thr kinase family,^189^ plays a crucial role in regulating cell proliferation, differentiation, and migration.^190^ The MAPK pathway consists of at least three cascade reactions involving three enzymes MAPK, MAPK kinase (MAPKK), and MAPK kinase kinase (MAPKKK).^191^ Each cascade reaction is initiated by specific extracellular signals and leads to the activation of a specific MAPK upon sequential activation of MAPKKK and MAPKK.^192^ The MAPK family is composed of three kinases: ERK, JNK, and p38 MAPK (MAPK14).^193^ The RAF–MEK–ERK pathway is the most representative MAPK signaling pathway, and its important upstream regulators include cell surface receptors such as RTKs, GPCRs, and integrins, as well as GTPases, Ras, and Rap.^194^ ERKs can translocate to the nucleus and phosphorylate different transcription factors, including Elk‐1, Sap‐1a, and c‐Myc, altering gene expression to promote growth, differentiation, or mitosis.^195^

According to several studies, MAPKs have been demonstrated to have significant implications in regulating bone density and bone homeostasis through their influence on the differentiation processes of osteoblasts and osteoclasts.^196^ First, mechanical stimuli can increase the expression of Focal adhesion kinase (Fak), which is an important member of integrin‐mediated signal transduction, thereby activating the Fak–MAPK pathway by activating Erk, Jnk, and p38–MAPK during distraction osteogenesis.^197^ In addition, the activation of the ERK, p38, and JNK signaling pathways can expedite osteoclast differentiation by modulating the activity of AP‐1, which serves as a critical regulator of osteoclast formation.^198^ In osteoblasts, TNF stimulates the activation of SHN3 through ERK MAPK‐mediated phosphorylation. Subsequently, phosphorylated SHN3 suppresses Wnt/β‐catenin signaling while increasing the expression of RANKL. Consequently, introducing a mutation in Shn3 that hinders its binding with ERK MAPK enhances bone formation in mice that overexpress human TNF‐α by boosting Wnt/β‐catenin signaling.^199^ In addition, the ERK signaling pathway plays a crucial role in regulating various phases of osteoblast differentiation through the phosphorylation of essential transcription factors, including RUNX2, and activation of transcription factor 4.^200^ Similarly, activation of the p38 signaling pathway facilitates the differentiation of osteoblasts through the phosphorylation of DLX5, OSX, and RUNX2.^201^ In addition, in a study by Xu et al.,^202^ endothelial progenitor cells were found to have a significant effect on BMSC differentiation and osteogenesis through the MAPK pathway. In accordance with prior research findings, the p38 signaling pathway has been shown to play a role in the regulation of ALP activity during the process of osteoblast differentiation.^203^ Moreover, this pathway is essential for the expression of BSP and OPN through the activation of p38MAPK–RUNX2 signaling.^204^ The absence of p38α in pre‐osteoblasts has been shown to lead to impaired osteoblast differentiation, as indicated by decreased levels of collagen 1, ALP, BSP, and OCN.^205^ Furthermore, Luo et al.^198^ discovered that the increased expression of disintegrin‐like and metalloproteinase with thrombospondin and MMPs through the activation of the ERK signaling pathway is significantly involved in the initial stages of OA progression.

In osteocytes, MAPK signaling is essential for regulating autophagy and protecting osteocytes from oxidative stress and other cytotoxic insults. It also responses to mechanical stimuli and interacts with other signaling pathways, such as the ERK, JNK, and p38–MAPK pathways, to maintain bone homeostasis^206^ (Figure 5).

### 3.8 AMPK signaling pathway

AMPK, a serine–threonine kinase,^207^ is a heterotrimeric complex consisting of an α‐subunit, a β‐subunit, and a γ‐subunit,^208^ of which the α‐subunit plays a catalytic role, while the β‐ and γ‐subunits play important roles in maintaining trimeric stability and substrate specificity.^209^ The AMPK signaling pathway functions as a detector of cellular energy levels and is triggered by elevated cellular AMP/ATP ratios resulting from metabolic stress, including interference with ATP production (e.g., glucose or oxygen deprivation) or accelerated ATP consumption (e.g., muscle contraction).^210^ Upon activation, AMPK simultaneously suppresses energy‐expending biosynthetic processes, including glycogen, protein and fatty acid synthesis, while stimulating ATP‐generating catabolic pathways such as glycolysis and fatty acid oxidation.^211^ In essence, AMPK regulates cellular energy allocation by suppressing protein synthesis and cell growth, as well as modulating cell cycle arrest by reducing the activity of mTOR, a protein that is overactive in many cancer cells.^212^ Consequently, AMPK has emerged as a promising target for the treatment of metabolic disorders and cancer.^213^ In addition, according to many studies, the activation of AMPK has the potential to influence the promotion of bone formation and bone density, indicating the significance of AMPK signaling as a crucial pathway in skeletal physiology.^209^

Initially, the regulatory impact of AMPK on osteoblasts primarily occurs via the activation of the osteoblast‐specific transcription factor RUNX2.^214^ Furthermore, AMPK can influence bone formation by promoting osteoblastogenesis through the down‐regulation of AGEs and the activation of IR deformation.^215^ Conversely, the AMPK signaling pathway suppresses RANKL by reducing the expression of peroxisome proliferator‐activated receptor gamma 1 (PPARγ1), NFATc1, PTH‐related protein (PTHrP), and mevalonate, consequently impeding the process of osteoclast formation.^216^ Notably, adipocytes and osteoblasts share a lineage with BMSCs as common progenitors.^217^ PPARγ2 inhibits osteoblast differentiation, promoting the differentiation of BMSCs into adipocytes,^218^ whereas the differentiation of BMSCs into osteoblasts is regulated by AMPK through the modulation of RUNX2 and a newly identified pathway, the Wnt/β‐catenin pathway.^219^ In addition, the mevalonate pathway, which is involved in the prenylation of regulatory proteins such as Ras and Rho GTPase, has been found to have a detrimental impact on bone tissue.^216^ AMPK has been shown to modulate the mevalonate pathway by inhibiting 3‐hydroxy‐3‐methylglutaryl‐coa reductase.^220^

Furthermore, AMPK signaling in osteocytes is vital for protecting against oxidative stress, regulating bone remodeling, and interacting with glucose metabolism. By modulating the expression of RANKL and SOST, AMPK helps maintain a balance between bone formation and resorption.^221^ Additionally, its role in enhancing OCN expression links bone metabolism with systemic glucose homeostasis, highlighting its importance in both skeletal and metabolic health^222^ (Figure 5).

### 3.9 Hh signaling pathway

The Hh signaling pathway consists of the Hh ligand, patched receptor (Ptch), smoothened receptor (Smo), suppressor of fused (Sufu), and the transcription factor glioma‐associated oncogene (Gli).^223^ There are three subtypes of Hh genes in mammals: Indian Hh, sonic Hh (Shh) and desert Hh, of which Ihh plays a major role in skeletal development. Ptch represents a transmembrane receptor consisting of 12 passes that interact with Hh ligands, encompassing two similar genes known as Ptch1 and Ptch2.^227^ Smo is a seven‐transmembrane protein that functions as a signal sensor and is inhibited by Ptch. Generally, the Hh protein relieves the inhibitory effect of Ptch on Smo by binding to Ptch on target cells, leading to Smo activation. Sufu is a negative regulator of the Hh pathway. However, activated Glis with a zinc finger structure dissociates from the Sufu‐containing repressor complex and contributes to the regulation of certain downstream target genes of cell‐active Hh signaling. Typically, Gli1 and Gli2 function primarily as transcriptional activators, whereas Gli3 acts as a repressor of Hh signaling.^224^

According to many studies, Hh signaling inhibits the differentiation of BMSCs into adipocytes while stimulating their differentiation into chondrocytes and osteoblasts, further maintaining bone homeostasis and promoting endochondral bone formation.^225^ In the process of embryonic development, the Hh signaling pathway triggers the upregulation of PTHrP in chondrocytes, thereby controlling the rate of chondrocyte hypertrophy within the developing growth plates.^8^ Apart from that, it is essential for the differentiation of osteoblasts through the regulation of RUNX2 expression. However, in postnatal bone remodeling, it has been shown that Hh signaling serves dual roles in regulating bone mass. A partial enhancement of Hh signaling due to Ptch1 haploinsufficiency leads to an increase in bone mass, while a widespread elevation of Hh signaling particularly in mature osteoblasts stimulates both bone formation and bone resorption. The non‐cell‐autonomous function of Hh signaling in the process of osteoclast differentiation is facilitated through the release of PTHrP from fully developed osteoblasts, leading to the stimulation of RANKL expression.^226^ The information presented indicates that Hh signaling in bones utilizes both intrinsic and extrinsic mechanisms to intricately oversee bone remodeling. In addition, the Hh signaling pathway also has key functions in synergistic cascades with other signaling pathways, such as the Wnt/β‐catenin, BMP, and PThrP pathways.^227^, ^228^ In osteocytes, the Hh pathway is integral for maintaining bone homeostasis and responding to mechanical stimuli. It plays a non‐cell‐autonomous role by affecting the differentiation and activity of other bone cells through the modulation of signaling molecules and pathways.^229^

In summary, once activated, the Hh pathway promotes the differentiation of stem cells to osteoblasts and the deposition of bone matrix and inhibits the apoptosis and destruction of osteoblasts through a complex of direct actions and interactions with other pathways. This is corroborated by current studies on osteoporosis, where inhibition of the Hh signaling pathway leads to inhibition of osteoblast proliferation and differentiation, thereby affecting bone formation and reducing bone density (Figure 5).

### 3.10 Notch signaling pathway

The Notch signaling pathway is a crucial intercellular communication mechanism playing a pivotal role in cell differentiation, proliferation and fate determination.^230^ The pathway includes four receptors, Notch1 to Notch4, which are transmembrane proteins capable of receiving external signals on the cell surface. The activation of Notch receptors is dependent on direct binding with ligands from two main families: the Jagged family (Jagged1 and Jagged2) and the Delta‐like family (including Delta‐like 1, Delta‐like 3, etc.). These ligands are also cell surface proteins that are expressed on neighboring cells and trigger Notch signal transduction. The binding of ligands to Notch receptors triggers a series of cleavage events, resulting in the release of the Notch intracellular domain (NICD) from the membrane. The NICD is a key executor of Notch signaling. The released NICD subsequently translocates to the cell nucleus, where it acts as a transcription factor to activate the transcription of specific genes. The primary target genes include the Hes families (hairy and Enhancer of split E(spl) homologs) and Hey families (Hes‐related proteins containing the YRPW motif).^231^ The expression of these genes affects cell proliferation, differentiation, and survival. Recent studies have emphasized the role of Notch signaling in regulating bone homeostasis, particularly its inhibitory effect on osteoblasts.^232^

The Notch pathway's activation hinders the differentiation of osteoblastic cells and results in impaired bone formation. Notch inhibits early osteoblast differentiation, preventing the maturation of matrix‐synthesizing cells, while in mature cells, it blocks further differentiation, causing an accumulation of dysfunctional osteoblasts. NICD binds to RUNX2, obstructing Bglap transactivation, and Hes and Hey proteins inhibit osteoblastogenesis by suppressing RUNX2 function.^233^, ^234^, ^235^ Additionally, in cells overexpressing Notch, cytosolic β‐catenin levels and Wnt‐3‐induced ALP activity are reduced. Hes1 complexes with Groucho and T‐cell‐specific factors, inhibiting the interaction between T‐cell factors and β‐catenin, which is essential for the transcription of Wnt‐dependent genes.^236^ Thus, osteoblastic differentiation was inhibited. Notch also regulates osteoblast differentiation and bone homeostasis via antiapoptotic actions. Jagged1 (JAG1), a notch activator, enhances the activation of antiapoptotic factor BCL‐2 and reduces the proapoptotic factor Caspase3 in osteoblasts.^237^ In osteocytes, the presence of Notch1 leads to specific outcomes, including the suppression of bone resorption through the upregulation of OPG and downregulation of SOST, ultimately resulting in increased activation of Wnt signaling.^231^ Moreover, Notch1 increases Wnt/β‐catenin activity in osteocytes by downregulating SOST and Dkk1 (Dickkopf Wnt signaling pathway inhibitor 1).^238^ There is a degree of overlap between Notch and Wnt activation in osteocytes. Notch receptors are induced in mice expressing a constitutively active β‐catenin mutant, and Notch further activates the Wnt/β‐catenin pathway in osteocytes, which creates a positive feedback loop.^233^

The roles of Notch1 and Notch2 in osteoclastogenesis and bone resorption are distinctly different. Stimulation of Notch1 receptors in pOCs leads to direct suppression of osteoclast formation.^239^ Concurrently, Notch1 activation in mature osteoblasts and osteoclasts stimulates OPG production, which hinders bone resorption.^240^ Conversely, Notch2 NICD interacts with NF‐κB, promoting the transcription of NFATc1, a key gene for osteoclastogenesis, thus enhancing osteoclastogenesis through both direct and indirect pathways. Furthermore, Notch2 activation in osteoblasts indirectly triggers RANKL, boosting osteoclastogenesis^241^ (Figure 5).

### 3.11 Ion channel signaling pathways

Cellular mechanotransduction plays a crucial role in the development and maintenance of bone tissue, with mechanical stimuli playing a key role in promoting bone formation and preventing bone loss.^242^ BMSCs, osteoblasts, osteocytes, and osteoclasts, which are the primary cell types found in bone tissue, respond to various mechanical cues such as matrix stiffness, fluid shear stress, tension, and compression across the lifespan of an individual.^243^ Physiological mechanotransduction activates relevant signaling pathways and maintains bone homeostasis. However, aberrant cellular mechanotransduction may lead to a variety of bone diseases, including osteoporosis and OA.^244^ Recent studies have revealed that certain ion channels, such as Piezo1, TMEM16A, P2X7, TRPV4, and other ion channels, which are closely related to skeletal homeostasis, play key roles in cellular mechanotransduction.^245^

#### 3.11.1 Piezo1

Piezo1 is a type of molecular receptor that mediates the perception of mechanical forces in the human body.^246^ It plays a crucial role in mechanotransduction within osteoblasts and osteocytes. Mechanical stimuli, such as compression and fluid shear stress, activate Piezo1 channels, inducing BMP2 expression and triggering intracellular calcium flux. This process promotes osteoblast differentiation by regulating the expression of bone‐related genes like RUNX2.^247^ Additionally, Piezo1 controls the expression of bone matrix proteins, including type II and type IX collagen, by regulating the intranuclear translocation of YAP1.^243^ In osteocytes, Piezo1 is involved in the Wnt1 and AKT signaling pathways. It promotes Wnt1 expression in osteocytes through the activation of YAP1 and TAZ.^248^ Furthermore, the activation of Piezo1 in osteocytes inhibits the expression of SOST via the Akt signaling pathway, thereby promoting bone formation.^249^

#### 3.11.2 Chloride channels: ClC‐7 and TMEM16A

Chloride channels play important roles in osteoclasts. During bone resorption, osteoclasts continue to secrete H^+^ ions into the lacunae and dissolve the bone matrix. In osteoblasts, there is a significant presence of chloride channel 7 (ClC‐7) in the ruffled membrane, which is created through the merging of vesicles containing H^+^‐ATPase. These vesicles release protons into the lumen. Destruction of ClC‐7 in mice leads to severe petrosalmia due to the inability of osteoclasts to secrete acid.^250^

Recently, studies have shown that the calcium‐activated chloride channel TMEM16A (also known as Anoctamin 1, ANO1) is another chloride channel related to bone resorption. Deletion of the TMEM16A gene can decreases H^+^ secretion, increases the intracellular Cl^−^ concentration and reduces bone resorption. In addition, TMEM16A physically binds to RANK and copromotes RANKL‐induced downstream signaling pathways.^251^

#### 3.11.3 P2X7

The purinergic P2X7 ion channel receptors are ATP‐regulated, cell‐surface, trimeric ligand‐gated cation channels regulating inflammatory responses and bone metabolism.^252^ Recent research has shown that the receptor is significantly involved in the anabolic reactions triggered by mechanical loading on bone. Additionally, in conjunction with the Panx1 hemi‐channel, it plays a crucial role in the initiation of bone remodeling in response to micro‐damage.^253^ In osteoblasts, P2X7 receptors play a role in the subsequent production and discharge of lysophosphatidic acid and prostaglandin E2 (PGE2) and in the stimulation of ERK1/2. This process facilitates osteoblast differentiation and the formation of bone tissue.^254^ In addition, P2X7 receptors are key factors in the inflammatory response; inflammatory factors promote the development of osteoporosis, and inhibition of P2X7 receptors reduces TNF‐α‐induced osteoclast differentiation.^255^

#### 3.11.4 TRPV4

The transient receptor potential (TRP) calcium channel is a prototypical mechanosensitive channel that plays a role in detecting diverse stimuli across a range of tissues and cellular environments.^256^ Among them, TRP vanilloid (TRPV) channel subfamily member 4 (TRPV4) regulates inflammation, mechanosensation, and energy homeostasis. In response to oscillatory fluid shear stress, TRPV4 channels induce calcium influx and activate CaMKII, which enhances cytoskeletal stiffness and affects osteoblast function by regulating genes such as OPN.^257^ TRPV4 mediates basolateral Ca^2+^ influx when Ca^2+^ oscillations are attenuated, especially in large osteoblasts.^258^ TRPV4‐mediated Ca^2+^ influx ensures the intracellular Ca^2+^ concentration, ensures NFATc1‐regulated gene transcription, and regulates terminal differentiation and activity of osteoblasts.^259^ In addition, fluid shear stress can activate NADPH oxidase 2‐generated reactive oxygen species (ROS) through the microtubule network, and ROS target the Ca^2+^ channel TRPV4, leading to Ca^2+^ inward flow. This pathway links fluid shear stress to ROS and Ca^2+^ signaling, leading to reduced SOST abundance in cultured osteoblasts.^260^

### 3.12 Noncoding RNA signaling pathways

Noncoding RNA refers to RNA that does not encode proteins, including microRNAs (miRNAs), long noncoding RNAs (lncRNAs), circular RNAs, and other RNAs with known functions.^261^ It is well recognized that miRNAs are small noncoding RNAs that can recognize cognate sequences and interfere with transcriptional, translational, or epigenetic processes.^262^ LncRNAs are defined as RNA transcripts >200 nucleotides that do not encode proteins.^263^ There is accumulating evidence that these RNAs are transcribed from the genome, and although they cannot encode proteins, they can exert their biological functions at the RNA level as regulatory factors, affecting various biological processes such as bone metabolism.^264^

In general, miRNAs can induce gene silencing by binding to mRNAs, while competing endogenous RNAs may modulate gene expression through competitive combining to miRNA response elements.^265^, ^266^ In contrast, both circRNAs and lncRNAs have miRNA‐binding sites, which bind to miRNAs in cells, thus counteracting the inhibitory effect of miRNAs on their target genes and thus increasing the level of target gene expression.^267^, ^268^

Some noncoding RNAs, such as lncRNAs ORLNC1,^269^ lncRNA‐POIR,^270^ miRNA‐433‐3p,^271^ circRNA‐0016624,^272^ and circRNA‐AFF4,^273^ have been shown to promote osteoblast differentiation by regulating substances associated with their differentiation. Moreover, some noncoding RNAs can also affect the differentiation of other cells, such as osteoclasts, thus promoting bone destruction.^274^, ^275^, ^276^ Notably, the corresponding biological functions of these noncoding RNAs need to be targeted by genes or pathways to produce indirect effects.

For instance, miRNA‐214, an important regulator of musculoskeletal metabolism and disease,^277^ can be expressed in human osteoporotic bone tissue to prevent osteoporosis by effectively regulating bone metabolism bidirectionally, promoting osteoblasts but inhibiting osteoclast activity, reducing bone loss, and effectively delaying osteoporosis pathology.^278^ Moreover, lncRNA KCNQ1OT1 can silence this gene to promote BMP2 expression to regulate osteogenic differentiation.^279^ Therefore, these RNAs play important regulatory roles in various signaling pathways through their interactions with each other, thus affecting the differentiation of osteoblasts and osteoclasts. Finally, they can regulate the balance of bone homeostasis.^280^

And noncoding RNAs have been implicated in many orthopedic diseases in which they play important roles.^281^, ^282^, ^283^, ^284^ For instance, lncRNA AC006064.4‐201 disrupts the stability of CDKN1B mRNA through interaction with PTBP1, thereby alleviating cartilage aging and protecting against OA.^285^ It has also been reported that the lncRNAs MALAT1,^286^ TUG1,^287^ and DANCR^288^ play different roles in OA by targeting different miRNAs. For instance, miR‐214‐3p can downregulate IKK‐β expression and lead to dysfunction of the NF‐κB signaling pathway, thus relieving the damage caused by OA.^289^

In addition, noncoding RNAs can also reach other cells via EVs and act as significant messengers in cellular crosstalk and biological activities.^30^ For example, EVs derived from BMSCs can deliver the lncRNA NEAT1 to relieve arthritis.^290^ EVs can be encapsulated by miR‐206,^291^ miRNA‐208a,^292^ the lncRNA PVT1,^291^ and other noncoding RNAs, and transported to osteosarcoma cells, leading to the proliferation, migration and invasion of cancer cells. However, EVs can regulate Wnt/β‐catenin signaling by decreasing TCF/LEF activity to further inhibit the proliferation and induce the apoptosis of osteosarcoma cells.^293^

## 4 THE ROLE OF BONE HOMEOSTASIS IN SKELETAL DISEASE

**FIGURE 6:**
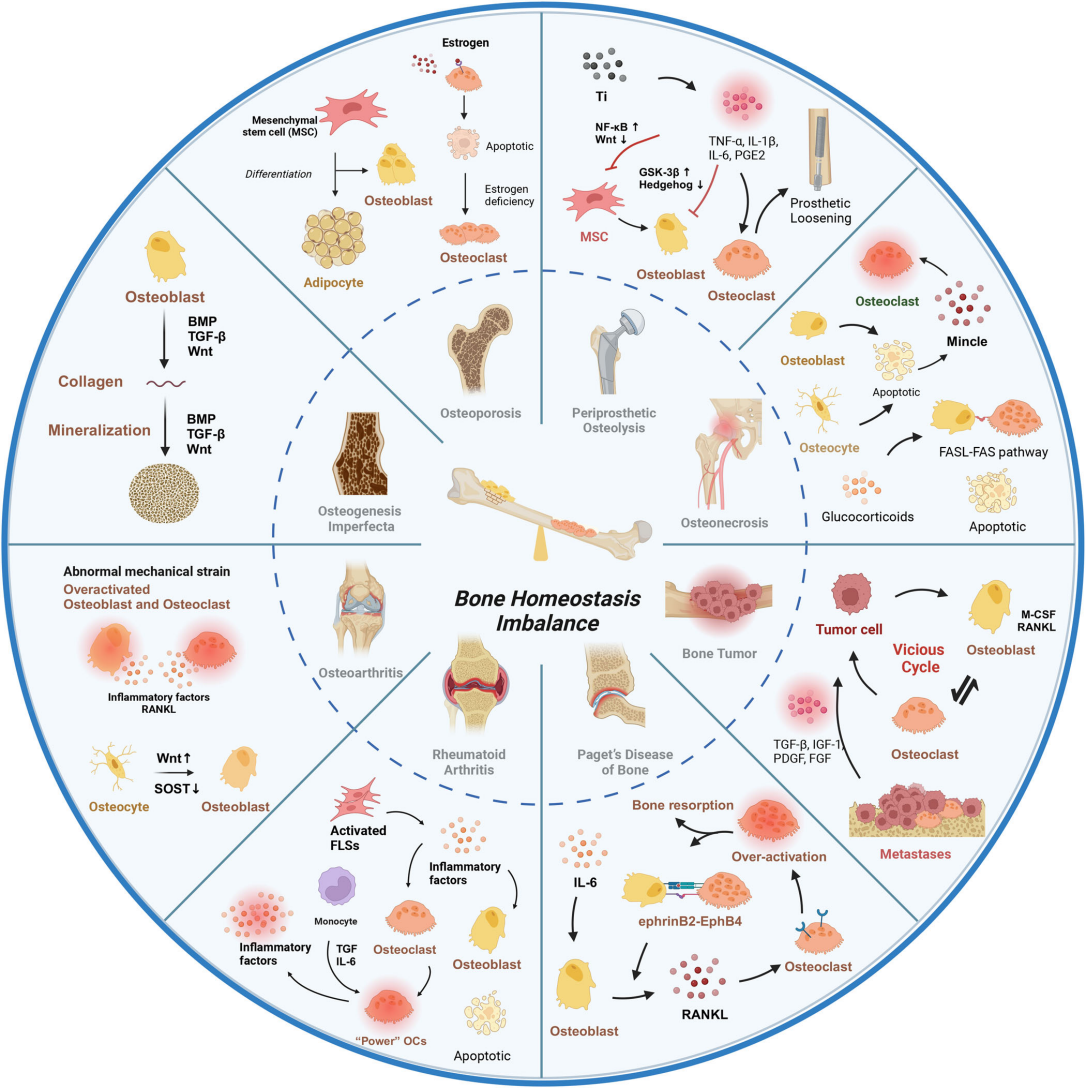
Overview of bone homeostasis disorders in skeletal diseases. (1) Osteoporosis: the main types of osteoporosis: postmenopausal osteoporosis and senile osteoporosis. Estrogen withdrawal leads to increased osteoclast activity, and senility decreases osteoblast differentiation and increases lipogenic differentiation. (2) Osteogenesis imperfecta: gene mutations and abnormal signaling pathways lead to impaired osteogenesis and mineralization. (3) Osteoarthritis: the interaction between stress changes and inflammatory environment activates dysfunction in osteoclast and osteoblast. (4) Rheumatoid arthritis: excessive activation of FLS with inflammatory factors is conducive to bone resorption by osteoclasts and impairs bone formation by osteoblasts, which leads to dysregulation of bone homeostasis and joint erosion. (5) Paget's disease of bone: due to the excessive activation of osteoclasts, the bone remodeling cycle is disrupted, resulting in excessive bone resorption and abnormal bone formation. (6) Bone cancer and metastases: bone metastatic cancer cells and osteoclasts enhance each other's activity, exacerbating the “vicious cycle” of bone degradation and tumor growth. (7) Osteonecrosis of the femoral head: it is caused by a variety of factors that affect bone health, such as the impacts of glucocorticoids, the role of the RANKL pathway, and the contributions of osteoclastogenic and osteoblastogenic cytokines. (8) Periprosthetic osteolysis: Ti wear particles will disrupt bone homeostasis by binding to immune cells and altering the activity of osteoblasts and osteoclasts (created with BioRender.com).

Since bone homeostasis is important contributor to bone development, repair, and remodeling, dysfunction of osteoblasts and osteoclasts is involved in the initiation and development of many diseases, such as OA, osteoporosis, OI, RA, PDB, bone cancer and metastases, ONFH, and PPO. It is becoming increasingly clear that the homeostasis of osteoblasts and osteoclasts plays important roles in the progression or remission of diseases. Here, we primarily discuss the pathology of various skeletal diseases and the biological effect of bone homeostasis on their occurrence and development. Moreover, we also introduce the various modulators and signaling pathways that affect skeletal cells either positively or negatively. Therapeutic interventions focused on the elements and controllers of signaling pathways in osteoblasts and osteoclasts exhibit significant promise and represent a promising avenue for addressing these conditions. (Figure 6)

### 4.1 Osteoporosis

Osteoporosis is a chronic disease characterized by reduced bone mineral density and structural deterioration, leading to an increased risk of fracture, functional limitations, and mortality.^7^ It has emerged as a major global health challenge, affecting people of all ages, sexes, and races, with the greatest impact on postmenopausal women and the elderly.^294^ Primary osteoporosis can generally be divided into postmenopausal and age‐related osteoporosis.

Menopause disrupts bone homeostasis due to estrogen withdrawal, which affects the activity of osteoblasts and osteoclasts. Physiologically, estrogen protects osteoblasts by preventing apoptosis and promoting their growth, maturation, and mineralization through various signaling pathways. Chang et al.^295^ reported that estrogen deficiency triggers NF‐κB activation in osteoblasts, inhibiting osteoblast function. For osteoclasts, estrogen depletion encourages osteoclastogenesis and bone resorption while deterring osteoclast apoptosis through various pathways. The absence of estrogen increases RANKL expression by the osteoblast lineage, including BMSCs, osteocytes, and bone lining cells, and diminishes OPG production, thereby intensifying osteoclast formation via the RANK–RANKL–OPG signaling system. Estrogen scarcity also curtails osteoclast apoptosis by inhibiting Fas/FasL signaling.^296^ Moreover, estrogen scarcity also boosts T‐cell activity and elevates the levels of inflammatory cytokines such as IL‐1, IL‐6, IL‐17, and TNF‐α, which indirectly stimulate osteoclast proliferation and differentiation by promoting RANKL and M‐CSF production.^297^ In conclusion, studies have highlighted the role of estrogen in protecting osteoblasts and its absence in promoting osteoclastogenesis.

In age‐related osteoporosis, there is a notable shift from osteogenesis to adipogenesis within BMSCs, guided by signaling pathways such as the ERK 1/2, TGF‐β, and BMP2/4 pathways.^298^ An increase in bone marrow adipocytes further exacerbates the development of osteoporosis, which not only impedes osteoblastogenesis but also favors osteoclastogenesis through the activation of PPARγ by free fatty acids (FFAs).^299^ Palmitic acid, a specific FFA, notably hinders osteoblast development and mineralization by suppressing the Wnt/β‐catenin and BMP2/RUNX2/SMAD pathways and induces apoptosis and autophagy dysfunction in osteoblasts.^300^ In addition, the interaction of PPARγ with specific promoter elements in hematopoietic stem cells promotes osteoclastogenesis,^301^ highlighting the complex interplay between factors that drive the progression of osteoporosis.

### 4.2 Osteogenesis imperfecta

OI is a hereditary skeletal disorder characterized by mutations in the COL1A1/COL1A2 genes, leading to increased bone fragility and skeletal deformities.^302^ Recent research has identified several signaling pathways, such as the Wnt, BMP, and TGF pathways, as significant contributors to the development of OI.^303^ For instance, mutations in the Wnt1 gene can disrupt the Wnt/β‐catenin pathway, impacting osteoblast growth, differentiation, and function.^304^ Studies by Grafe et al.^305^ have shown that modulating TGF‐β signaling in an OI mouse model can correct bone abnormalities and improve lung function. Gene ontology analysis of human samples has highlighted the upregulation of SMAD phosphorylation in OI patients, with TGF‐β identified as a critical activation signal through genomic enrichment and pathway analyses.^306^ FAM46A, a member of the nucleotidyltransferase folding protein superfamily, interacts with SMAD and promotes the transcription of BMP target genes, with mutations in FAM46A linked to autosomal recessive OI inheritance.^7^ Additionally, BMP1 plays a crucial role in cleaving the C‐terminal propeptide of procollagen types I, II, and III, and defects in this process have been implicated in OI pathogenesis.^4^

### 4.3 Osteoarthritis

OA is a degenerative condition of the joints marked by the gradual breakdown of articular cartilage, subchondral sclerosis, osteophyte development, varying levels of synovial inflammation, and meniscus degeneration. Persistent pain, swelling, deformity, limited joint function, and reduced mobility impose significant financial and emotional burdens on OA patients.^307^ In OA, the subchondral bone experiences remodeling, with simultaneous increases in bone resorption and formation.^203^ Abnormal mechanical strain disrupts osteoblast metabolism, leading to higher levels of IL‐6, PGE2, MMP, and RANKL, and lower levels of OPG. IL‐6 and PGE2 promote osteoclast formation by reducing OPG secretion and increasing RANKL production in osteoblasts or by elevating RANK expression in osteoclasts. PGE2 also triggers IL‐6 release, which in turn increases PGE2 secretion by osteoblasts.^308^ This creates a positive feedback loop between PGE2 and IL‐6 signaling, enhancing osteoclast differentiation by modulating the RANK/RANKL/OPG pathway.^309^ Additionally, heightened mechanical stress causes osteocytes to influence osteoblast mineralization by increasing Wnt protein production and decreasing SOST secretion. In vitro studies indicate that osteoclastic bone resorption significantly raises active TGF‐β1 levels in OA subchondral bone, promoting osteoblast‐driven bone formation through the SMAD2/3 pathway in advanced OA.^7^ Consequently, increased osteoblast activity results in spatial remineralization and osteosclerosis in late‐stage OA. Furthermore, TGF‐β can elevate neurofactors in OA via the ALK5–SMAD2/3 pathway and may contribute to joint pain in OA patients.^310^

### 4.4 Rheumatoid arthritis

RA is a systemic autoimmune disease characterized by a complex interplay of cellular activities and signaling pathways that leads to the deterioration of joints.^311^ Synovial inflammation and irreversible bone destruction are prominent features of RA that lead to an imbalance in bone homeostasis.^312^ Overactivated fibroblast‐like synoviocytes (FLSs) increase the expression of proangiogenic factors such as IL‐6 and chemokines and cause an imbalance between γδTreg and γδT17 cells.^313^ This environment causes acidic and hypoxic conditions in arthritic joints, which fosters the activation of osteoclasts and the apoptosis of osteoblasts, contributing to bone degradation.^314^ In detail, acidic and hypoxic conditions diminish the osteoblast production of ALP and impede mineralization. Moreover, hypoxia interferes with Wnt signaling by blocking β‐catenin transduction, and inflammatory factors such as TNF‐α disrupt this pathway further by inducing the overexpression of DKK‐1 in synovial fibroblasts.^315^ TNF‐α also negatively affects the synthesis of collagen type I, ALP activity, and the expression of OCN in vitro.^7^ In addition, peripheral blood monocytes (PBMCs) are thought to be the major source of osteoclasts in inflammatory environments, and osteoclasts derived from PBMCs show an enhanced bone resorption capacity.^316^ Notably, the synovial fluid of RA patients contains mononuclear cells that express osteoclast‐related genes and can form osteoclasts spontaneously, indicating a heightened osteoclastogenic potential in RA.^7^ These developments are further amplified by inflammatory cytokines such as TNF‐α and IL‐6, which activate the JAK–STAT signaling pathway and promote the differentiation of PBMCs into osteoclasts, leading to increased bone resorption and bone destruction.^317^

### 4.5 Paget's disease of bone

PDB is a chronic focal disorder characterized by disturbed bone homeostasis, leading to asymmetric skeletal deformities. These deformities result from excessive bone resorption by abnormally large osteoclasts and disorganized bone formation by osteoblasts.^318^, ^319^ Pagetic osteoclasts, characterized by an increased number of nuclei, exhibit heightened responsiveness to osteoclastogenic factors, including RANKL and 1,25‐dihydroxyvitamin D3. This heightened responsiveness not only boosts their bone resorption capacity but also increases their resistance to apoptosis.^7^, ^320^ In response to the activity of these osteoclasts, coupling factors are released, increasing the number of immature osteoblasts at resorption sites. Notably, these immature osteoblasts produce more RANKL than their mature counterparts, exacerbating bone formation.^321^ Additionally, individuals with PDB exhibit elevated levels of IL‐6, which promotes RANKL expression on osteoblasts and stromal cells, potentially leading to osteoclast hyperactivation.^322^ This localized increase in RANKL is critical for disease progression. Although less is known about osteoblasts in the PDB, emerging research suggests that osteoclast‐osteoblast communication plays a vital role. For instance, the measles virus nucleocapsid protein can stimulate osteoclasts to upregulate IL‐6 and IGF‐1, enhancing EphrinB2–EphB4 coupling and thereby stimulating bone formation by osteoblasts.^323^, ^324^ Furthermore, pagetic phenotypes may boost osteoclast‐derived IGF‐1 expression, which, in turn, triggers additional RANKL production in osteocytes. This increase in RANKL production is a key factor in the development of pagetic lesions.^325^ In conclusion, disordered osteoclast and osteoblast activity together contribute to the development of PDB.

### 4.6 Bone tumors

Bone is a frequent site of metastasis for many cancers, particularly breast and prostate cancers, aside from primary bone tumors like osteosarcoma. Cancer cells facilitate bone degradation by inducing osteoclast‐mediated bone resorption, leading to substantial structural damage and severe morbidity. This process results in significant pain, fractures, and reduced quality of life for patients.^326^ Bone is a frequent site of metastasis for many cancers, particularly breast and prostate cancers, aside from primary bone tumors like osteosarcoma. Cancer cells facilitate bone degradation by inducing osteoclast‐mediated bone resorption, leading to substantial structural damage and severe morbidity. This process results in significant pain, fractures, and reduced quality of life for patients.^327^ Tumor‐derived M‐CSF and RANKL on osteoblasts, BMSCs, and immune cells bind to c‐fms and RANK on pOCs, initiating their differentiation and activation. Consequently, cancer cells stimulate osteoclasts to break down bone tissue, releasing growth factors (TGF‐β, IGF‐1, PDGF, and FGF) stored within the bone matrix and enhancing tumor growth.^328^ The interaction between cancer cells and the bone microenvironment creates a vicious cycle and induces bone tumor growth and bone metastasis.^329^ RANKL secreted by osteoblasts attracts cancer cells expressing RANK and promotes their migration within the bone microenvironment.^107^ Furthermore, osteoclasts contribute to this cycle by releasing factors that promote tumor growth and metastasis. Zuo and Wan^330^ found that PDL1 on tumor cells can bind to PD‐1 on osteoclasts, affecting the function of osteoclasts and further promoting tumor growth. Anti‐PDL1 treatment inhibited bone metastasis in breast cancer and melanoma and did so by inhibiting osteoclast formation and enhancing the immune response within the bone. In addition, another study showed that PD‐1 blockade inhibited osteoclast formation and bone cancer pain in mice.^331^ These findings suggest that although PD‐L1 is expressed on tumor cells primarily to inhibit T‐cell activity and help tumor cells evade immune surveillance, it also plays a significant role in bone metastasis and associated pain.

### 4.7 Osteonecrosis of the femoral head

ONFH is a prevalent orthopedic clinical condition marked by the demise of bone and marrow cells. This condition can be attributed to a range of factors including hip trauma, corticosteroid administration, alcohol misuse, hemoglobin disorders, bone marrow transplants, chemotherapy, and radiation exposure.^332^ ONFH results in pain, restricted mobility, joint collapse, and subsequent development of secondary OA.^333^ Glucocorticoids have a multifaceted impact, directly causing apoptosis of osteoblasts and osteocytes, extending the lifespan of osteoclasts, and inducing the apoptosis of endothelial cells.^7^ These actions contribute collectively to femoral head ischemia and bone marrow changes, illustrating the detrimental effects of corticosteroids on bone integrity.^334^ The induction of osteonecrosis by glucocorticoids in animal models has been attributed to GSK3β‐mediated osteoblast apoptosis, highlighting a specific pathway through which corticosteroids may exert their harmful effects.^335^ Necrotic osteocytes release macrophage C‐type lectin (Mincle) that induces osteoclast formation through ITAM‐based calcium signaling, leading to OxPhos and increased bone loss.^7^ In addition, osteoclasts and osteoblasts may undergo apoptosis after prolonged treatment with glucocorticoids through the FASL/FAS pathway. In corticosteroid‐associated osteonecrosis, corticosteroids can stimulate bone loss through their action on the RANK/RANKL/OPG signaling system. Several potential signaling pathways are involved in osteocyte apoptosis mitigation, such as the mitochondrial, MAPK, PI3K/Ak, Wnt/β‐catenin, and HIF‐1 signaling networks.^7^

### 4.8 Periprosthetic osteolysis

PPO, a critical complication after artificial joint replacement, jeopardizes prosthesis longevity and outcomes and increases treatment costs.^336^ Titanium implants generate wear particles over time due to friction, disrupting bone homeostasis by engaging immune cells and altering osteoblast and osteoclast activity. These particles provoke macrophage activation and inflammatory cytokine release (IL‐6, PGE2, IL‐1β, TNF‐α), driving osteoclastogenesis and jeopardizing BMSC osteogenic differentiation, which contributes to osteolysis and aseptic prosthesis loosening.^337^ Wear particles affect osteogenic differentiation through activating the NF‐κB signaling pathway and reducing the involvement of the Wnt/β‐catenin signaling pathway in BMSC activation of the ERK signaling pathway.^338^ Moreover, Ti particles inhibit the osteogenic differentiation of osteoblasts via the BMP/SMAD signaling pathway.^339^ Furthermore, we found that titanium particles lead to an aberrant increase in GSK‐3β activity, which inhibits the Hh/Gli1 signaling pathway, ultimately leading to an inhibition of the osteogenic process.^7^ Furthermore, we demonstrated that up‐regulation of SIRT3 expression enhanced the osteogenic differentiation of PPO289 by decreasing NLRP3 inflammasome expression via the GSK‐3β/β‐catenin signaling pathway.^7^ Wang et al.^340^ found that Netrin‐1 promotes autophagy induced by Ti particles and enhances osteoblast formation via the ERK1/2 signaling pathway.

## 5 TARGETED THERAPIES FOR SKELETAL DISEASES

### 5.1 Anabolic agents

#### 5.1.1 Teriparatide

Teriparatide, a recombinant human PTH (1−34) analog, is commonly used as an anabolic medication to help patients with osteoporosis avoid fractures.^341^ Intermittent low‐dose teriparatide treatment was demonstrated to stimulate osteoblastic activity, mostly through the stimulation of Wnt signaling, inhibition of DKK‐1, and production of IL‐11.^342^ A phase 4 clinical trial showed that teriparatide is a promising treatment for menopausal osteoporosis (NCT01293292).^343^ However, teriparatide increases both bone formation and bone resorption, which results in reduced cortical bone volume and thickness, particularly in long bones with greater proportions of cortical bone.^341^, ^344^ A phase 4 clinical trial showed that 1 year of teriparatide treatment did not significantly reduce bone erosion in RA patients (NCT01400516).^345^ In addition, due to the risk of osteosarcoma, teriparatide use is limited to those at a very high risk of fracture.^346^

#### 5.1.2 Abaloparatide

Abaloparatide, also known as PTHrP1–34, represents a synthetic version of the human PTH analog. It has been identified that PTH1R exists in at least two distinct conformations, named R0 and RG.^347^ Abaloparatide preferentially binds to the RG conformation of PTH1R, similarly to PTH (1–34), which recruits the Gs protein, triggering a temporary spike in cellular cAMP levels. This differs from ligand interactions at the R0 site, where cAMP responses are notably sustained.^348^ Due to its selective binding affinity, abaloparatide facilitates sharper and shorter signaling pathways compared with PTH (1–34), leading to increased bone‐anabolic effect with reduced bone resorption. This attribute is anticipated to surpass teriparatide in fostering bone anabolism. Consequently, abaloparatide promotes more transient signaling, leading to a greater with less enhancement of bone resorption than that of PTH (1–34). Thus, it is expected to induce a stronger anabolic effect than teriparatide. Clinical outcomes from a phase three study (NCT01343004) have demonstrated that abaloparatide significantly decreases the risk of new vertebral fractures by 86% and other types of nonvertebral fractures by 43% within an 18‐month trial period.^349^ Furthermore, abaloparatide promoted an increase in the expression of serum bone formation markers at an early age, indicating its antifracture properties and safety in long‐term and severe osteoporosis (NCT01657162).^350^

### 5.2 Antiresorptive agents

#### 5.2.1 Denosumab

Denosumab, a human monoclonal antibody that neutralizes RANKL, plays a critical role in managing bone‐related conditions by blocking the RANK/RANKL/OPG signaling pathway.^351^ This inhibition effectively prevents the formation, function, and survival of osteoclasts, making denosumab a potent treatment for osteoporosis. Notably, in a phase 3 clinical trial (NCT00089791), administering denosumab biannually was shown to significantly reduce fracture risk in women with osteoporosis.^352^ Further research into the efficacy of denosumab extends to erosive hand OA. A phase 2a clinical trial (NCT02771860) demonstrated that denosumab significantly lowers the development of new erosive joints compared with a placebo, demonstrating its potential to modify the structure of erosive hand OA.^353^ Additionally, osteoporosis patients with RA who were treated with denosumab, calcium and vitamin D, experienced a notable increase in BMD, underscoring its therapeutic benefits.^354^ Additionally, its efficacy extends to treating malignant bone metastases, where it not only aids in managing the disease but also exhibits direct or indirect antitumor effects in both preclinical models and clinical settings.^355^

#### 5.2.2 Bisphosphonates

Bisphosphonates, a class of compounds like pyrophosphate, have a strong inhibitory impact on osteoclasts and are therefore potent antiresorptive agents.^356^, ^357^ This class of drugs, which includes alendronate, risedronate, ibandronate, and zoledronate, is extensively used in clinical settings to treat joint and bone illnesses such as multiple myeloma, advanced osteoporosis, bone metastatic cancer, PDB, and RA.^358^ Although multiple studies have shown that bisphosphonates are effective in the treatment of osteoporosis (NCT03005678 and NCT00453492), their long‐term usage may be related to osteonecrosis; therefore, caution should be exercised when using these medications (NCT01526915).^359^

#### 5.2.3 Fresolimumab

Fresolimumab (GC1008) is a pan‐TGFβ neutralizing antibody^360^ in clinical trials for OI and cancer. It effectively inhibits all three isoforms, particularly TGFβ1.^361^ As there is no specific therapy for OI, drugs initially developed for osteoporosis are being tested for their effectiveness in treating adults with OI (NCT03064074).^362^

#### 5.2.4 Paroxetine

Paroxetine is a selective inhibitor of 5‐hydroxytryptamine reuptake, which is extensively employed for the treatment of depression, anxiety disorders, and Alzheimer's disease.^363^ Recent studies have revealed that paroxetine can suppress pyroptosis and curtail the creation of osteoclasts by blocking the NF‐κB signaling pathway. This indicates its potential therapeutic benefits for individuals suffering from OA.^7^ Furthermore, the applicability of paroxetine in treating RA is currently under investigation, as evidenced by ongoing research (NCT06231745).

### 5.3 Anabolic–antiresorptive agents

#### 5.3.1 Romosozumab

Romosozumab is a humanized monoclonal antibody targeting SOST, a protein secreted by osteocytes that plays a critical role in the regulation of bone homeostasis. SOST inhibits the Wnt signaling pathway in osteoblasts.^364^, ^365^ By neutralizing SOST, romosozumab enhances the Wnt pathway, leading to increased bone formation and, to a lesser extent, decreased bone resorption.^366^, ^367^ Clinical studies have demonstrated that treatment with romosozumab significantly improves BMD at key sites such as the spine and hip and reduces the risk of vertebral fractures in postmenopausal women with osteoporosis (NCT00896532, NCT01575834).^368^, ^369^ Moreover, while romosozumab triggers an early and transient boost in bone formation, it more importantly results in a sustained decrease in bone resorption, thereby reducing overall bone turnover.^370^ Romosozumab may be more effective than other drugs for bone formation. In a phase 3 clinical trial, romosozumab showed superior efficacy in increasing BMD compared with other treatments, such as alendronate and teriparatide (NCT01631214).^371^ Alongside romosozumab, other anti‐SOST monoclonal antibodies, such as Blosozumab (NCT02109042) and BPS804 (NCT01406548), are currently under development, indicating a growing interest and recognition of the therapeutic potential of targeting SOST in bone disease management.

#### 5.3.2 Adalimumab

TNF‐α antagonists, such as adalimumab, etanercept, infliximab, and golimumab, can promote a reverse signal that blocks the NF‐κB pathway.^372^, ^373^ TNF‐α is an important pathological mediator in both OA and RA. It can induce bone resorption directly by inducing osteoclastogenesis or indirectly by affecting RANKL, OPG, and PG production by osteoblasts.^374^ Therefore, numerous studies have been conducted to establish that adalimumab can inhibit these pathways to treat OA and RA (NCT00686439, NCT02035800, and NCT01585064).^375^

#### 5.3.3 Menatetrenone

Menatetrenone (vitamin K2, VK2) is a fat‐soluble vitamin. Recent studies have found that VK2 may improve osteoporosis in individuals with type 2 diabetes by inhibiting ferritin deposition through activation of the AMPK/SIRT1 signaling pathway.^376^ In a phase 4 clinical trial, menatetrenone increased OCN secretion and gamma‐carboxylation, with significant but modest increases in bone resorption and bone formation indices (NCT00548509).

#### 5.3.4 Curcumin

Over the last half‐century, extensive studies have demonstrated that curcumin (diferuloylmethane), a component of the yellow spice turmeric, may alter various cell signaling pathways.^377^ Curcumin has shown favorable results in terms of its antioxidant, anti‐inflammatory, and anticancer properties.^378^, ^379^ Therefore, it has been developed for the treatment of several cancers. However, studies have also begun to explore the use of curcumin for the treatment of RA because it is also a potent inhibitor of Notch1 (NCT00752154).

### 5.4 Anti‐inflammatory agents

#### 5.4.1 Sulfasalazine

The IKK complex, which consists of IKKα and IKKβ as kinase subunits and IKKγ as a regulatory subunit, is activated upon stimulation of the conventional NF‐κB pathway. Since IKKα and IKKβ are essential components of the NF‐κB pathway, blocking them essentially obstructs the entire pathway.^380^ Sulfasalazine, a disease‐modifying antirheumatic medication developed for RA and other autoimmune diseases, directly inhibits IKKα and IKKβ by blocking ATP binding.^381^, ^382^ Many nonsteroidal anti‐inflammatory drugs, inhibitors of the NF‐κB pathway, can inhibit ATP binding to IKKβ, thereby preventing the activation and translocation of NF‐κB to the nucleus.^383^ Furthermore, patients suffering from symptomatic OA in the knee might be treated with intra‐articular injection of SAR113945, which is an IKK inhibitor.^384^ However, this drug has not been further validated in a three‐phase clinical trial (NCT01113333, NCT01463488).

#### 5.4.2 Tofacitinib

Tofacitinib (CP‐690,550) is an innovative oral inhibitor of Janus kinase, currently being studied as a specific immunomodulator and therapy for modifying disease in RA. It impedes RANKL‐mediated osteoclastogenesis by blocking the activation of STAT3 and NF‐κB pathways.^385^ Tofacitinib primarily targets JAK1 and JAK3, with some effect on JAK2 and tyrosine kinase 2 (TYK2). In addition, tofacitinib can restore the γδ Treg/γδT17 cell balance, which effectively ameliorates RA progression.^386^ In individuals with active RA, tofacitinib monotherapy has been linked to a decrease in RA signs and symptoms and better physical function (NCT00814307).^387^ The combination of tofacitinib and iguratimod has been found to alleviate RA and secondary osteoporosis in a mouse model.^388^ However, a clinical study revealed that tofacitinib promotes bone marrow adipocyte differentiation in RA patients (NCT04175886).^389^ Consequently, more research is necessary to explore tofacitinib's impact on osteoporosis.

#### 5.4.3 Baricitinib

Baricitinib is an orally administered small‐molecule JAK inhibitor that is used to treat moderate‐to‐severe RA.^361^ Compared with placebo and adalimumab, baricitinib significantly improved clinical outcomes in RA patients who had not respond well to methotrexate treatment (NCT01710358).^390^

#### 5.4.4 Tocilizumab

Tocilizumab, which can be used in combination with methotrexate, is an IL‐6 receptor antagonist approved in many countries around the world.^391^ It can be used to treat adults with moderately to severely active RA by inhibiting IL‐6 and blocking the jak/stat signaling pathway.^392^ The long‐term efficacy and safety of tocilizumab have been established through extensive clinical experience in patients with both early and severe RA (NCT03781310, NCT02031471).^393^ (Table 1)

**TABLE 1:** Targeted therapeutic drugs for skeletal diseases in clinical trials.

Agent type	Therapeutic target	Agent	Source	Phase	Enrollment	Disease
Anabolic	PTH1R	Teriparatide	NCT03002428	Phase 3	181	OP
			NCT01293292	Phase 4	19	OP
			NCT01400516	Phase 4	26	RA
		Abaloparatide	NCT03710889	Phase 3	23	OP
			NCT01343004	Phase 3	2463	OP
Antiresorptive	RANKL	Denosumab	NCT00089791	Phase 3	7808	OP
			NCT01799798	Phase 2	10	OI
			NCT02771860	Phase 2	100	OA
			NCT01973569	Phase 3	679	RA
			NCT02470091	Phase 2	56	OS
			NCT00286091	Phase 3	1435	BM
			NCT02299817	Phase 2	20	PPO
	TGFβ1	Fresolimumab	NCT03064074	Phase 1	11	OI
	OCs	Alendronate	NCT03005678	Phase 4	140	OP
		Risedronate	NCT00453492	Phase 4	246	OP
		Ibandronate	NCT00683163	Phase 2/3	44	OP
		Zoledronate	NCT03183557	Phase 2	100	OP
	NF‐κB	Paroxetine	NCT06231745	Phase 3	100	RA
Anabolic–antiresorptive	TNF‐α	Adalimumab	NCT00686439	Phase 1/2	20	OA
			NCT02035800	Phase 4	120	RA
	Sclerostin	Romosozumab	NCT01588509	Phase 1	60	OP
			NCT01575834	Phase 3	7180	OP
			NCT04545554	Phase 1	25	OI
		Blosozumab	NCT02109042	Phase 1	15	OP
		Setrusumab (BPS804)	NCT01406548	Phase 2	44	OP
			NCT03118570	Phase 2	112	OI
			NCT05125809	Phase 2/3	219	OI
	Notch‐1	Curcumin	NCT00752154	Phase 1	40	RA
	AMPK	Menatetrenone	NCT00548509	Phase 4	131	OP
Anti‐inflammatory	NGF	Tanezumab	NCT02697773	Phase 3	698	OA
	IKKα/IKKβ	Sulfasalazine	NCT01113333	Phase 1	40	OA
			NCT01463488	Phase 1	24	OA
	NF‐κB	Cyclophosphamide	NCT02693210	Phase 2	161	RA
	NF‐κB/AP‐1	Corticosteroids	NCT01724268	Phase 3	80	RA
	JAK1/3	Tofacitinib	NCT00814307	Phase 3	200	RA
	JAK1/2	Baricitinib	NCT01710358	Phase 2	120	RA
	IL‐6/JAK1	Tocilizumab	NCT03781310	Phase 4	80	RA
			NCT02031471	Phase 3	57	RA

Abbreviations: BM, bone metastases; NGF, nerve growth factor.; OA, osteoarthritis; OCs, osteoclasts; OI, osteogenesis imperfecta; ONFH, osteonecrosis of the femoral head; OP, osteoporosis; OS, osteosarcoma; RA, rheumatic arthritis.

*Source*: Clinical Registration website https://www.clinicaltrials.gov.

## 6 CONCLUSION AND PERSPECTIVE

A deep comprehension of the intricate regulatory processes governing signaling pathways in bone maintenance is essential for the effective management and mitigation of bone‐related disorders. In this review, we focused on bone homeostasis and its signaling pathway mechanisms in various bone disease states. By modulating these signaling pathways, either through activation or inhibition, we can uncover new pathways for bone disease research and treatment.

Although current drugs targeting bone homeostasis, such as denosumab, teriparatide, and tofacitinib, have been very successful in the clinic, nonspecific targets can result in severe side effects. Recent years have seen a significant widening of research horizons in signaling pathways across multiple fields. Researchers have investigated novel targeted treatment strategies, including the use of peptides (WP9QY and OP3‐4) that block the interaction between RANKL and RANK^394^, ^395^ and the development of an innovative vaccine (mRANKL‐MT3) designed to activate LGR4. These approaches aim to suppress osteoclast activation by interfering with the RANK‐RANKL‐OPG signaling pathway.^396^ Chen et al.^397^ used the highly selective JAK3 inhibitor Z583 for treating RA, which avoids adverse events and side effects. In addition, Xu et al.^398^ established BMP2 immune complexes (BMP2‐ICs) that can bind to osteoblast‐ and osteoclast‐lineage cells through the BMP2‐conjugated domain and Fc domain, respectively, facilitating communication between osteoblasts and osteoclasts and activating EphrinB2–EphB4 signaling to promote osteoblastogenesis and inhibit osteoclastogenesis. In addition, some traditional natural Chinese medicines have been found to target signaling pathways and are also used to treat skeletal diseases.^399^ This diversification has enabled the repurposing of drugs originally designed for nonorthopedic conditions for bone disease treatment, leveraging their role in the regulation of bone homeostasis. To overcome the cardiac ischemia induced by the SOST inhibitor romosozumab, Wang et al.^400^ developed a novel SOST inhibitor, Apc001PE, that promotes bone formation in OI mice by targeting loop3 to recognize recombinant SOST and SOST in the serum of OI patients without increasing cardiovascular risk.

However, the specificity and safety of these therapeutics warrant further in‐depth analysis. Ongoing investigations into the cellular and molecular foundations of bone homeostasis hold promise for pioneering enhanced therapeutic strategies for tackling bone‐related diseases. To decipher the complex molecular mechanisms and interactions within signaling pathway networks that control bone homeostasis, it is imperative to thoroughly explore the convergence points within these pathways. Such exploration could be key to unlocking novel and effective strategies for bone health management.

## AUTHOR CONTRIBUTIONS

Zebin Wu, Wenming Li, and Kunlong Jiang conceived and drafted the manuscript. Zhixiang Lin and Chen Qian made the figures. Mingzhou Wu, Ning Li, and Yu Xia made the tables. Hongtao Zhang, Jiaxiang Bai, and Haixiang Xiao edited and revised the manuscript. Dechun Geng supervised and revised the manuscript. All the authors have read and approved the final manuscript.

## CONFLICT OF INTEREST STATEMENT

The authors declare no conflict of interest.

## ETHICS STATEMENT

No ethical approval was required for this study.

## Data Availability

Not applicable.
